# Loss of ST8SIA6 is associated with enhanced MUC16-Integrin β4 signaling and aggressive phenotypes in colorectal cancer

**DOI:** 10.1016/j.bbrep.2026.102757

**Published:** 2026-08-25

**Authors:** Pei-Chun Shih, Yung-Kuo Lee, Ching-Cheng Hsu, Chung-Hsien Lin, Ngoc Uyen Nhi Nguyen, Hsin-Pao Chen, Wei-Ling Lin, Chuan-Fa Chang

**Affiliations:** a Institute of Basic Medical Science, College of Medicine, National Cheng Kung University, Tainan, 70101, Taiwan; b Medical Education and Research Center, Laboratory of Research, Kaohsiung Armed Forces General Hospital, Kaohsiung, 80284, Taiwan; c Division of Cardiology, Department of Internal Medicine, Morsani School of Medicine, University of South Florida, Tampa, FL, USA; d Division of Colon and Rectal Surgery, Department of Surgery, E-DA Hospital, I-Shou University, Kaohsiung, 82445, Taiwan; e Department of Medical Laboratory Science and Biotechnology, College of Medicine, National Cheng Kung University, Tainan, 70101, Taiwan; f Center of Infectious Disease and Signaling Research, College of Medicine, National Cheng Kung University, Tainan, 70101, Taiwan; g University Center of Bioscience and Biotechnology, National Cheng Kung University, Tainan, 701401, Taiwan; h Department of Pathology, National Cheng Kung University Hospital, College of Medicine, National Cheng Kung University, Tainan, Taiwan

**Keywords:** Colorectal cancer, Metastasis, ST8SIA6, MUC16, Integrin β4, Glycosylation

## Abstract

Colorectal cancer (CRC) remains a leading cause of cancer-related mortality, underscoring the need to better understand tumor-intrinsic mechanisms underlying aggressive disease progression. Aberrant glycosylation is increasingly recognized as a key regulator of cancer cell behavior; however, the functional contribution of α2,8-sialylation to CRC remains poorly defined. Here, we investigated the role of the α2,8-sialyltransferase (ST8SIA6) in CRC progression and its impact on mucin-mediated signaling. Analysis of human CRC tissue arrays showed that ST8SIA6 expression was reduced in higher-stage tumors. ST8SIA6 silencing enhanced migration and invasion and was associated with EMT-like molecular changes. Using Siglec-7-based lectin enrichment and proteomic analysis, we identified MUC16 as a prominent Siglec-7-reactive glycoprotein associated with ST8SIA6. Loss of ST8SIA6 reduced Siglec-7 reactivity of MUC16 and increased its cell-surface localization. Increased membrane-associated MUC16 was accompanied by enhanced association with integrin β4 and increased Src and FAK phosphorylation. Blockade of integrin β4 attenuated Src/FAK activation and suppressed CRC cell migration, supporting a functional role for this axis. In vivo, ST8SIA6-deficient CRC cells exhibited increased lung tumor colonization following tail-vein injection in xenograft models, accompanied by elevated membrane MUC16 and increased MUC16-integrin β4 colocalization. Consistent with these findings, human CRC specimens from advanced stages exhibited increased MUC16 expression and integrin β4 colocalization compared with early-stage tumors. Together, these findings support a model in which loss of ST8SIA6 enhances MUC16-integrin β4 signaling and contributes to aggressive CRC phenotypes through increased MUC16 membrane localization and integrin β4-dependent signaling. These findings identify a previously unrecognized glycosylation-associated signaling axis that warrants further investigation as a biomarker and potential therapeutic target in colorectal cancer.

## Highlights

• ST8SIA6 expression is reduced in advanced colorectal cancer.
• ST8SIA6 loss is associated with enhanced CRC migration, invasion, and lung colonization.
• ST8SIA6 loss is associated with increased membrane localization of MUC16.
• Membrane-associated MUC16 is associated with integrin β4 colocalization and Src/FAK phosphorylation.
• ST8SIA6-MUC16-integrin β4 signaling is associated with aggressive CRC phenotypes.

## Graphical abstract

**Figure:**
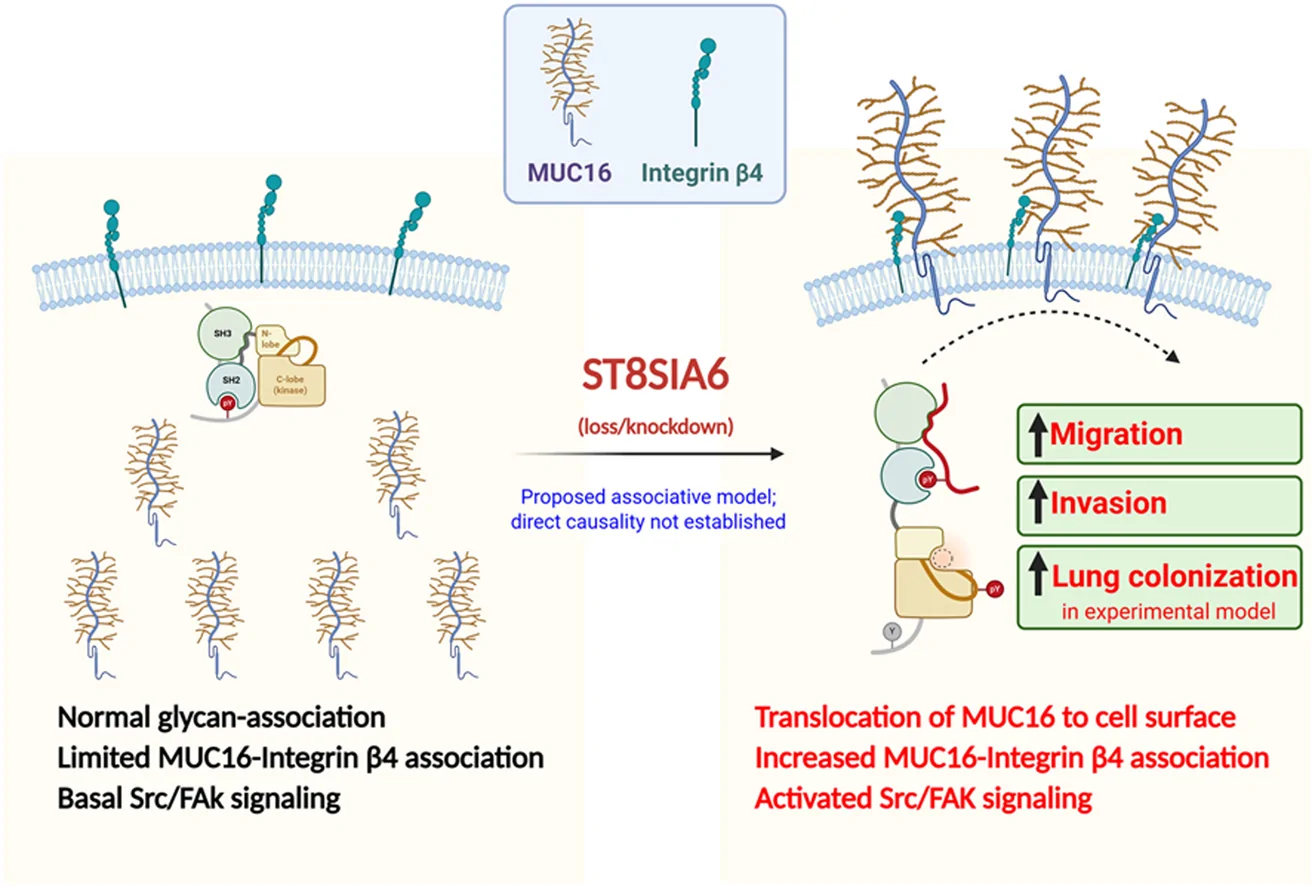

## 1 Introduction

Colorectal cancer (CRC) remains a leading cause of cancer-related mortality worldwide, with metastatic dissemination accounting for the majority of disease-associated deaths [1]. Despite advances in surgical techniques and systemic therapies, a substantial proportion of patients either present with metastatic disease at diagnosis or develop distant metastases during treatment [2,3]. Current prognostic stratification and therapeutic decision-making remain insufficient to accurately identify tumors with high metastatic potential, underscoring the need to define tumor-intrinsic mechanisms that drive aggressive progression and may be exploited for therapeutic intervention [4,5].

Aberrant protein glycosylation is increasingly recognized as a hallmark of cancer that modulates cell-cell interactions, receptor signaling, and metastatic behavior [6]. Altered sialylation patterns, in particular, influence tumor cell adhesion, migration, and survival by reshaping the molecular landscape of the cancer cell surface [7]. These processes are regulated by sialyltransferases, which catalyze the addition of sialic acids to glycoproteins and glycolipids. While sialylation has been extensively studied in colorectal cancer, the functional role of α2,8-linked sialylation in CRC progression remains comparatively underexplored [[8], [9], [10], [11], [12], [13]].

ST8SIA6 is a member of the α2,8-sialyltransferase family and is known to generate disialylated glycan structures on selected glycoproteins [11,14,15]. Prior studies have implicated ST8SIA6 in diverse cancer-associated processes, including immune modulation through Siglec engagement and regulation of glycoprotein trafficking [11,[16], [17], [18]]. Notably, the role of ST8SIA6 appears context-dependent, with reports describing tumor-promoting functions in certain malignancies and reduced expression associated with disease progression in others. In colorectal cancer, bioinformatic and clinical analyses have suggested that ST8SIA6 expression is decreased in advanced-stage tumors and correlates with unfavorable outcomes [12,19]; however, the tumor-intrinsic mechanisms underlying this association remain poorly defined.

Mucins are heavily glycosylated transmembrane or secreted proteins that play critical roles in epithelial homeostasis and cancer progression [[20], [21], [22]]. Among them, MUC16 (CA125) is a well-established tumor-associated mucin and clinical biomarker in several malignancies [22,23]. In CRC, elevated MUC16 expression has been associated with tumor progression and adverse disease features [24]. Beyond its diagnostic value, emerging evidence suggests that MUC16 contributes to tumor cell migration and invasion by interacting with cell surface receptors and intracellular signaling pathways [25]. Nevertheless, how post-translational glycosylation regulates MUC16 localization and function in CRC remains largely unknown.

Integrin β4 is a transmembrane adhesion receptor that promotes tumor cell survival, motility, and metastatic progression through activation of Src and focal adhesion kinase (FAK) signaling [26,27]. Aberrant integrin β4 signaling has been linked to aggressive behavior in multiple epithelial cancers, including colorectal cancer, and represents a potentially actionable therapeutic target [[28], [29], [30]]. Whether mucin-glycosylation-dependent mechanisms in CRC regulate integrin interactions remains unclear.

In this study, we investigated the role of ST8SIA6 in colorectal cancer progression and explored its impact on MUC16-associated signaling. By integrating analyses of human CRC tissues, in vitro functional assays, and in vivo metastasis models, we show that loss of ST8SIA6 is associated with enhanced migration and invasion in vitro and increased lung colonization in an experimental metastasis model. Our findings support a model in which reduced ST8SIA6 expression alters Siglec-7 reactivity of MUC16 and promotes its membrane localization, and is associated with increased MUC16-integrin β4 interaction and Src/FAK activation. These results identify a previously unrecognized glycosylation-associated pathway that may contribute to metastatic progression and offer potential translational opportunities for biomarker development and therapeutic targeting in colorectal cancer.

## 2 Materials and methods

### 2.1 Antibodies

The following commercial antibodies were used: AKT (GeneTex, USA, Catalog Number: GTX640258, Clone Name: HL2915), Phospho-AKT (Abcam, USA, Catalog Number: ab81283, clone Name: EPR2347), ERK1/2 (GeneTex, USA, Catalog Number: GTX50560), Phospho-ERK1/2 (Cell Signaling Technology, USA, Catalog Number: 4370, Clone Name: D13.14.4 E), Src (GeneTex, USA, Catalog Number: GTX111182), Phospho-Src (GeneTex, USA, Catalog Number: GTX24846), E-Cadherin (Cell Signaling Technology, USA, Catalog Number: 3195, Clone Name: 24E10), N-Cadherin (Cell Signaling Technology, USA, Catalog Number: 13116, Clone Name: D4R1H), FAK (Cell Signaling Technology, USA, Catalog Number: 3285), Phospho-FAK (Cell Signaling Technology, USA, Catalog Number: 3283), Integrin β4 (GeneTex, USA, Catalog Number: GTX108780). For immunohistochemistry (IHC), anti-ST8SIA6 (Abcam, Catalog Number: ab99067) and anti-MUC16 (Abcam, Catalog Number: ab110640) were used at a 1:200 dilution.

### 2.2 Cell culture

The HCT116 CRC cell line was purchased from the Food Industry Research and Development Institute (Hsinchu, Taiwan) and maintained in a growth medium consisting of McCoy's 5 A medium (Gibco, Thermo Fisher Scientific, USA) supplemented with 100 units/mL penicillin, 100 μg/mL streptomycin, and 10% fetal bovine serum (HyClone, Cytiva, USA) at 37°C and 5% CO_2_. T5088 colorectal cancer cells were maintained in a growth medium consisting of F12 medium supplemented with 100 units/mL penicillin, 100 μg/mL streptomycin, and 10% fetal bovine serum (Hyclone, USA) at 37°C and 5% CO_2_.

### 2.3 CRC tissue samples

This study was approved by the Institutional Review Board of E-DA Hospital (IRB No: EMRP22107 N). Following patient consent, clinical CRC tissue samples were obtained from the biobank of the E-DA Hospital in Kaohsiung, Taiwan. A colorectal adenocarcinoma tissue array (CO1501), comprising 70 colorectal adenocarcinoma tissue biopsies, was purchased from Biomax (Maryland, USA). These tissues were derived from Asian individuals, including 11 patients at stage I, 36 at stage II, 19 at stage III, and 4 at stage IV. Details on the patient information for the tissue microarray are listed in Table S1. After overnight fixation in 4% formalin, these tissues were placed in the embedding cassette and subjected to dehydration by sequential immersion in a graded ethanol and xylene for 1 h each, before being embedded in paraffin for 2 h at 58°C. After embedding, the tissues were sectioned into 5 μm-thick slices, mounted on slides, and stained.

### 2.4 Immunohistochemistry (IHC)

IHC staining of the tissue sections was performed using an HRP Labeling Kit with DAB Brown (TAHC03D, BioTNA, Taiwan) according to the manufacturer's instructions. Briefly, formalin-fixed paraffin-embedded tissue sections were deparaffinized and rehydrated, followed by antigen retrieval with an enzyme digester (TA00H01, BioTNA, Taiwan). Endogenous peroxidase activity was blocked by adding a hydrogen peroxide block solution (containing 3% hydrogen peroxide) for 10 min. Then, a blocking solution containing PBS and 0.5% bovine serum albumin (BSA) was added to the tissue sections, which were incubated for 30 min at RT before incubation with primary antibodies for 1 h at the same temperature. After washing three times in PBS, a Mouse/Rabbit Probe HRP Labeling system (containing polymer-conjugated horseradish peroxidase) was applied to bind with the primary antibodies for 30 min at RT. After labeling, the slides were incubated with DAB Chromogen for 10 min to visualize the antibody signals. Finally, hematoxylin (3,801,560, Leica, Germany) and eosin (3,801,600, Leica, Germany) were used for counterstaining. The slides were dehydrated in ascending alcohol solutions and observed via an optical microscope (TissueFaxs system, Tissue Gnostics, USA). The percentage of ST8SIA6 or MUC16 staining was quantified using ImageJ by measuring the DAB-positive area in 40 fields per sample.

### 2.5 Lentivirus-based RNAi knockdown of ST8SIA6

RNAi-mediated knockdown of ST8SIA6 was carried out as previously described [19]. The shRNA targeting ST8SIA6 (TRCN0000436740) was obtained from the RNA Technology Platform and Gene Manipulation Core of Academia Sinica (Taipei, Taiwan) and constructed via the pLKO_TRC005 vector. The specific sequence targeted by the ST8SIA6 shRNA was 5′-CCTGCTGTGATGCTGTTCAAA-3′, whereas the target sequence for the vector control was 5′-TGTTCGCATTATCCGAACCAT-3'. The lentiviral vector for sh-ST8SIA6 was constructed by the RNAi Core of the Clinical Medicine Research Center at National Cheng Kung University Hospital (Tainan, Taiwan). To establish stable sh-*ST8SIA6* cell lines, cells were transfected with lentivirus containing 8 μg/mL polybrene (Sigma‒Aldrich, St. Louis, MO, USA) and incubated at 37°C overnight. Infected cells were selected with 2 μg/mL puromycin (Sigma‒Aldrich, St. Louis, MO, USA). The efficiency of ST8SIA6 knockdown was assessed by Western blotting (WB).

### 2.6 Total RNA preparation and real-time quantitative PCR (RT‒qPCR)

Total RNA was extracted from cells via TRIzol reagent (Invitrogen, Carlsbad, USA) and RNA extraction kits (Qiagen, USA). The concentration and quality of the RNA were verified via a NanoDrop ND-1000 UV‒Vis spectrophotometer (NanoDrop Technologies, USA). cDNA was synthesized from 2 μg of RNA via MMLV reverse transcriptase (Promega, USA) according to the manufacturer's instructions. A StepOnePlus™ Real-Time PCR System (Applied Biosystems, USA) was used to perform RT-qPCR. For RT-qPCR, cDNA was thoroughly mixed with sense and antisense primers and SYBR Green Master Mix (Thermo Fisher Scientific, USA). All the genes were normalized to the GAPDH internal control. The primer sequences for ST8SIA6: forward: CGGCAAGCAGAAGAATATG; reverse: GCTTTCCACCTCGTAACTC. The primer sequences for GAPDH: forward: CTCCTCCACTTTGACGCTG; reverse: TCCTCTTGTGCTCTTGCTGG.

### 2.7 Western blotting analysis

Cellular total proteins were isolated with RIPA buffer supplemented with a cocktail of protease inhibitors (Cat# S8820, Sigma‒Aldrich, USA). Total protein concentrations were quantified using a BCA protein assay kit (Cat#23225; Thermo Fisher Scientific, USA) and measured using a spectrophotometer (Cat#U-2800 A; Hitachi, Tokyo, Japan). The membrane proteins were extracted via a membrane fraction extraction kit (Cat#ab284937; Abcam, Cambridge, England). Proteins were separated on an 8-10% polyacrylamide gel and transferred to polyvinylidene difluoride (PVDF, 0.45 μm) membranes (Millipore, USA). The membranes were then incubated with specific primary antibodies at 4°C overnight, followed by incubation with secondary antibodies conjugated with HRP at RT for 1 h. Then, the membranes were subjected to enhanced chemiluminescence (ECL) (Millipore, USA), and the signals were detected via a chemiluminescence imaging system (Fujifilm LAS 4000, Fujifilm, Japan). Blots were optimized for exposure and loading.

### 2.8 Wound healing assay

The cells (5 × 10^5^/mL in each chamber) were seeded in two chambers of the culture insert (ibid, USA) to reach full confluency. After incubation for 24 h at 37°C and 5% CO_2_, the culture insert was removed, and micrographs were taken at 0 and 24 h via an optical microscope. The areas of migrated cells were measured using ImageJ. Experiments were performed at least in triplicate with three biological replicates.

### 2.9 Transmigration assay

A transwell apparatus (8-μm pore-size polycarbonate membrane, 6.5-mm diameter; Corning, Inc., Corning, NY) was used to determine cell transmigration. CRC cells (5 × 10^4^ cells/well) were seeded in a transwell insert containing 100 μL of serum-free medium, and 500 μL of medium supplemented with 10% fetal bovine serum was added to the lower chamber. After incubation for 18 h at 37°C, nontransmigrated cells in the insert were gently removed with cotton swabs. The migrated cells on the opposite side of the membrane were fixed in methanol for 30 min at RT and then stained with 1% crystal violet for 1 h. The migrated cells were visualized via an optical microscope, and the number of migrated cells was quantified. Experiments were performed at least in triplicate with three biological replicates.

### 2.10 Lectin pull-down and protein identification

According to the manufacturer's instructions, one mg of total cell lysate was incubated with Siglec-7 agarose beads overnight at 4°C. The pulled-out proteins were separated via electrophoresis. The gel was dehydrated with acetonitrile, rehydrated in distilled water, and dehydrated in acetonitrile. Then, the samples were rehydrated in trypsin/50 mM ammonium bicarbonate buffer at 4°C for 1 h and then covered with 50 mM ammonium bicarbonate for overnight digestion. The mixture was centrifuged, and the peptides were extracted with 5% formic acid in acetonitrile. The protein mixture was loaded on a liquid chromatography-mass spectrometry plate and dried at room temperature.

### 2.11 Immunoprecipitation

The cells were lysed in coimmunoprecipitation (co-IP) lysis buffer (20 mM Tris, pH = 7.5, 120 mM NaCl, 2 mM EDTA, 0.2% NP-40, 10% glycerol, and protease inhibitors) and incubated with the target antibodies overnight at 4°C. After incubation, the protein and antibody complexes were incubated with protein A/G agarose beads (Cat# ab193262; Abcam, Cambridge, England) for 4 h. The complex was then subjected to immunoblotting as described.

### 2.12 Flow cytometry

The cells were detached with trypsin (GeneDireX, Inc., Taiwan), washed in PBS, and incubated with primary antibody at 4°C for 1 h. Then, the cells were incubated with a fluorescence-conjugated secondary antibody at 4°C for 30 min. After rinsing with PBS, flow cytometry data were analyzed on a FACSCalibur™ flow cytometer (BD Biosciences, USA). Data acquisition was carried out by using FlowJo.

### 2.13 Immunofluorescence

The cells were seeded on glass coverslips for cell staining and grown at 37°C for 24 h. After rinsing with PBS, the cells were fixed with 4% paraformaldehyde at 4°C for 30 min. For tissue section staining, 5 μm-thick sections were used. Autofluorescence was diminished via an autofluorescence quencher (Biotium, Fremont, CA, USA). Samples from the cell lines and tissue sections were incubated with PBS containing 5% BSA at RT for 1 h, followed by incubation with primary antibodies at 4°C overnight. The samples were subsequently incubated with fluorescence-conjugated secondary antibodies for 1 h at RT. The nuclei were stained with DAPI. Fluorescence images were obtained via a fluorescence microscope (Olympus FV1000).

### 2.14 Animals and ethics

The animal study protocols followed the guidelines of the National Laboratory Animal Center (NLAC) and the Laboratory Animal Center of National Cheng Kung University Medical College, and all the projects were approved by the Institutional Animal Care and Use Committee (IACUC Approval No. 111208). Nonobese diabetic/severe combined immunodeficient (NOD/SCID) mice (RRID:IMSR_RJ: NOD-SCID) were obtained from the Laboratory Animal Center of National Cheng Kung University (Tainan, Taiwan). The mice used in the experiments were 6-8 weeks old. CRC cells (1 × 10^6^) were intravenously injected via the tail vein to assess lung colonization. The mice were sacrificed after 6 weeks, and the lung tissues were harvested to investigate nodule formation. For the subcutaneous tumor model, CRC cells (1 × 10^6^) were mixed with Matrigel (50% v/v final concentration, Invitrogen, USA) and subcutaneously injected into the right flank of NOD/SCID mice as previously described [31]. After 4 weeks of tumor growth, the mice were sacrificed, and the tumor tissues were harvested for histological analysis.

### 2.15 Bioinformatics analysis

Gene expression and survival data for colorectal cancer patients were analyzed using the Gene Expression Profiling Interactive Analysis (GEPIA, http://gepia.cancer-pku.cn/) [32], the Human Protein Atlas (HPA, https://www.proteinatlas.org/) [33], and the Cancer Genome Atlas Program (TCGA, https://www.cancer.gov/ccg/research/genome-sequencing/tcga) web servers, which draw on GEPIA, HPA, and TCGA datasets [12].

### 2.16 Statistics

All the data are presented as the means ± standard deviations (SDs) or means ± standard errors of the means (SEMs). All experiments were independently repeated at least three times. The statistical analysis was performed via GraphPad Prism 9 software (Boston, MA, USA) and SPSS 23.0 (SPSS, Inc., Chicago, IL, USA).

Data distribution was assessed before statistical testing. For normally distributed data, two-group comparisons were performed using Student's t-test, and multi-group comparisons were performed using one-way ANOVA followed by Tukey's post hoc test. For datasets that did not meet parametric assumptions, two-group comparisons were performed using the Mann-Whitney *U* test, whereas multi-group comparisons were analyzed using the Kruskal-Wallis test followed by Dunn's multiple-comparison test. The statistical test used for each experiment is indicated in the corresponding figure legend. *P* < 0.05 was considered statistically significant.

## 3 Results

### 3.1 ST8SIA6 expression is reduced in advanced colorectal cancer

**Fig. 1:**
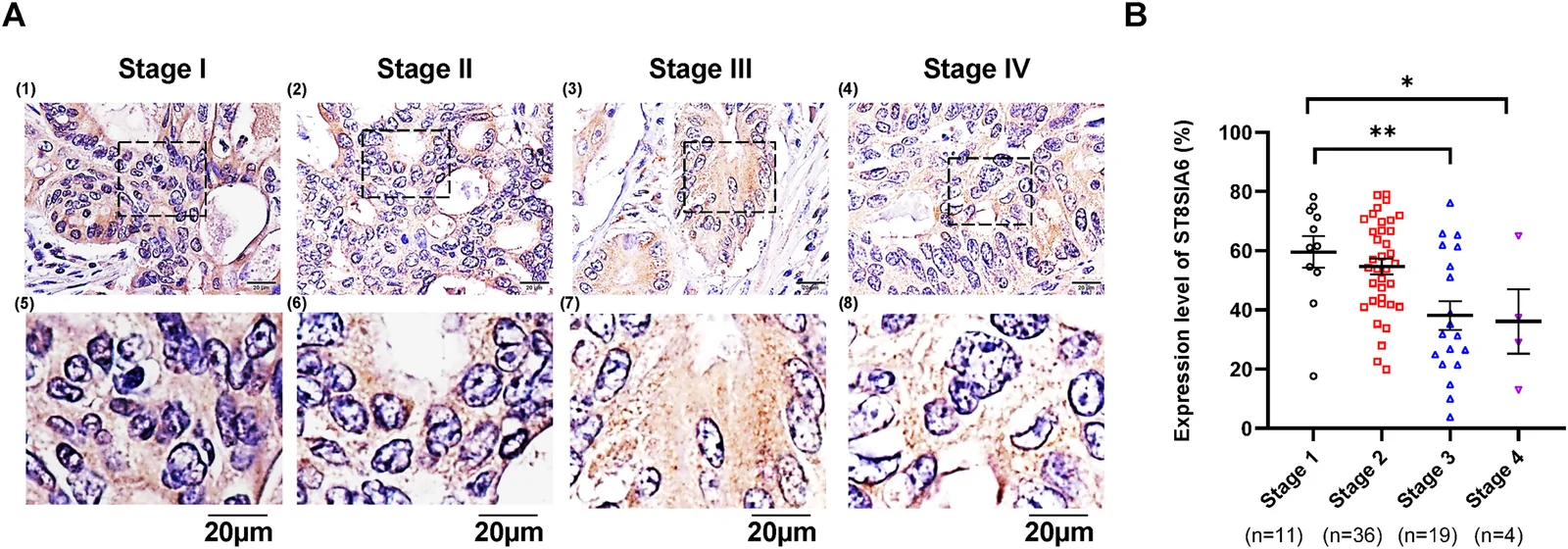
**Tissue array analysis shows decreased ST8SIA6 expression in the advanced stage of CRC. A.** Representative IHC images of ST8SIA6 expression in a human CRC tissue array. The lower row (5)-(8) shows enlarged images of the frame area corresponding to the upper row (1)-(4). Scale bars: as indicated in figure. B. The percentage of ST8SIA6 staining in each stage was quantified. Data are mean ± SEM. *p < 0.05, **p < 0.01 determined by Kruskal-Wallis test followed by Dunn's multiple-comparison test.

Our previous multiple gene database analysis revealed that ST8SIA6 expression is decreased in late-stage CRC [12]. To further verify this observation in clinical samples, ST8SIA6 IHC staining was performed on a tissue array comprising 70 CRC tissue biopsies from Asian individuals at stages I to IV. IHC analysis revealed lower levels of ST8SIA6 in more advanced stages of CRC (stages III and IV) than in early stages (stage I) (Fig. 1A and B), suggesting that ST8SIA6 was downregulated during CRC progression. Although stage IV samples were limited in number, a consistent reduction in ST8SIA6 expression was observed across advanced stages. In addition, our findings were comparable with the survival analysis of colon and rectum adenocarcinoma in HPA (Figs. S1 and S2) [12].

### 3.2 Loss of ST8SIA6 enhances migratory and invasive phenotypes in colorectal cancer cells

**Fig. 2:**
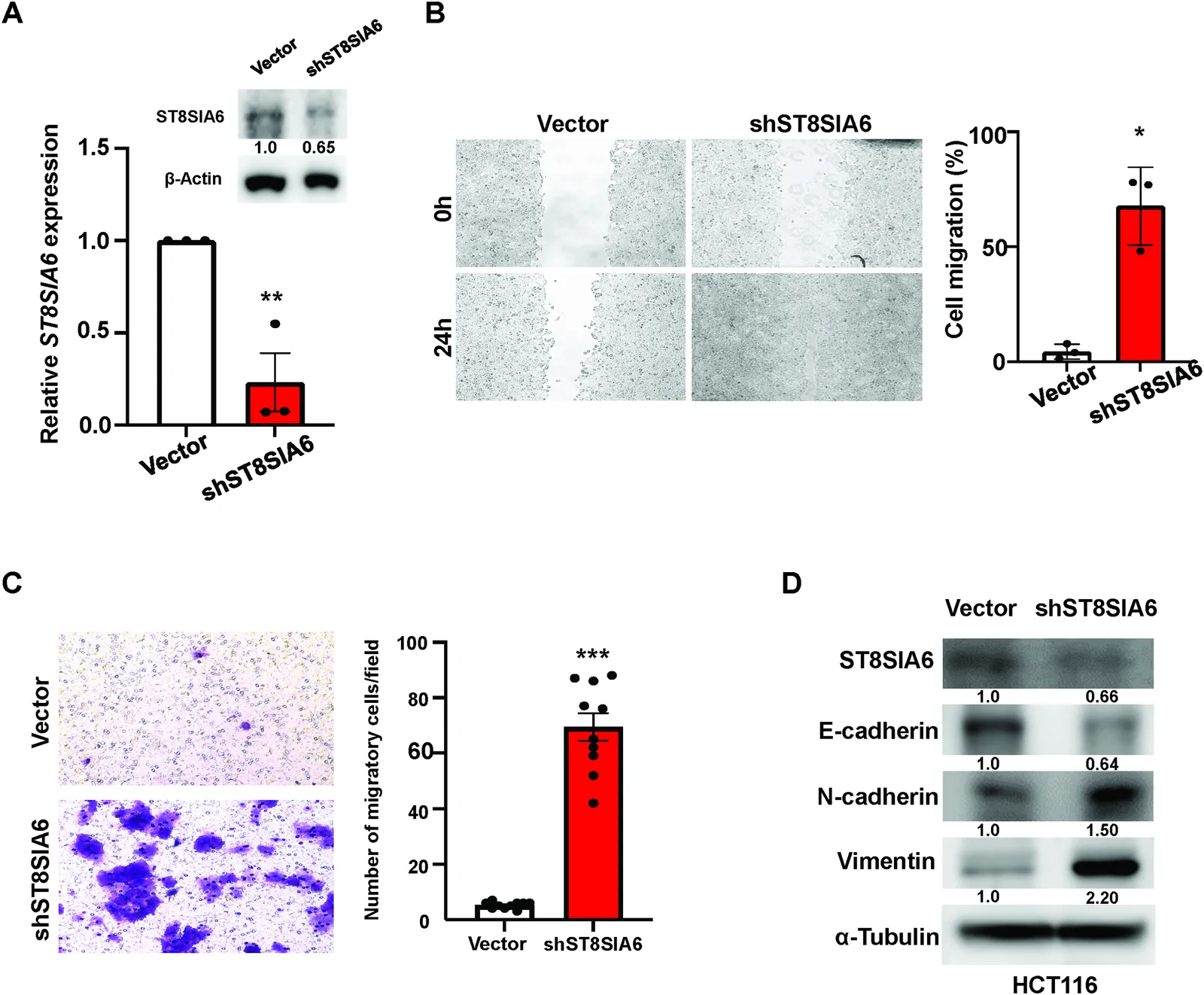
**ST8SIA6 reduction enhances CRC cell motility. A.** RT-qPCR and Western blot analysis of ST8SIA6 expression in HCT116 and T5088 cells transfected with control vector or sh-*ST8SIA6*, using β-Actin as a loading control. Data are mean ± SD, **p < 0.01 determined by Student's t-test. **B.** Wound healing assay in HCT116 and T5088 cells transfected with vector or sh-*ST8SIA6*. Representative light-microscope images at 0 h and 24 h, and quantification of the migrated cell area are shown. Original magnification: ×4. Data are mean ± SD, *p < 0.05 determined by Student's t-test. **C.** The transwell assay in HCT116 and T5088 cells transfected with vector or sh-*ST8SIA6*. Representative images of crystal violet staining and quantification of the migrated cells are shown. Original magnification: ×100. Data are mean ± SD. ***p < 0.001, determined by Student's t-test. **D.** Representative Western blot images of EMT-like markers (E-cadherin, N-cadherin, Vimentin) in HCT116 and T5088 cells transfected with vector or sh-*ST8SIA6*, using α-Tubulin as a loading control. Representative Western blot images and densitometric quantification are shown. n = 3 biological replicates.

To examine the role of ST8SIA6 in CRC progression, we knocked down ST8SIA6 expression in the CRC cell lines HCT116 and T5088. The downregulation of ST8SIA6 expression was confirmed by RT-qPCR and WB analysis (Fig. 2A and Fig. S3). The wound healing assay demonstrated a marked increase in HCT116 migration following ST8SIA6 silencing (Fig. 2B). ST8SIA6 knockdown modestly enhanced migration/invasion in T5088 cells (Fig. S1B and S1C), consistent with more potent effects in HCT116. Additionally, we performed a transwell assay to assess transmigration. The results consistently demonstrated significantly increased invasive potential in both CRC cells transfected with sh-*ST8SIA6* (Fig. 2C and Fig. S1C). We evaluated the protein levels of epithelial-mesenchymal transition (EMT)-like markers. The results revealed that E-cadherin expression was reduced, whereas N-cadherin and vimentin expression were increased in both ST8SIA6-knockdown cells compared with the vector control cells (Fig. 2D and Fig. S1D). Taken together, these data suggest that reduced ST8SIA6 expression is associated with enhanced migratory and invasive behavior in CRC cells.

### 3.3 MUC16 is identified as a Siglec-7-reactive glycoprotein affected by ST8SIA6 knockdown

**Fig. 3:**
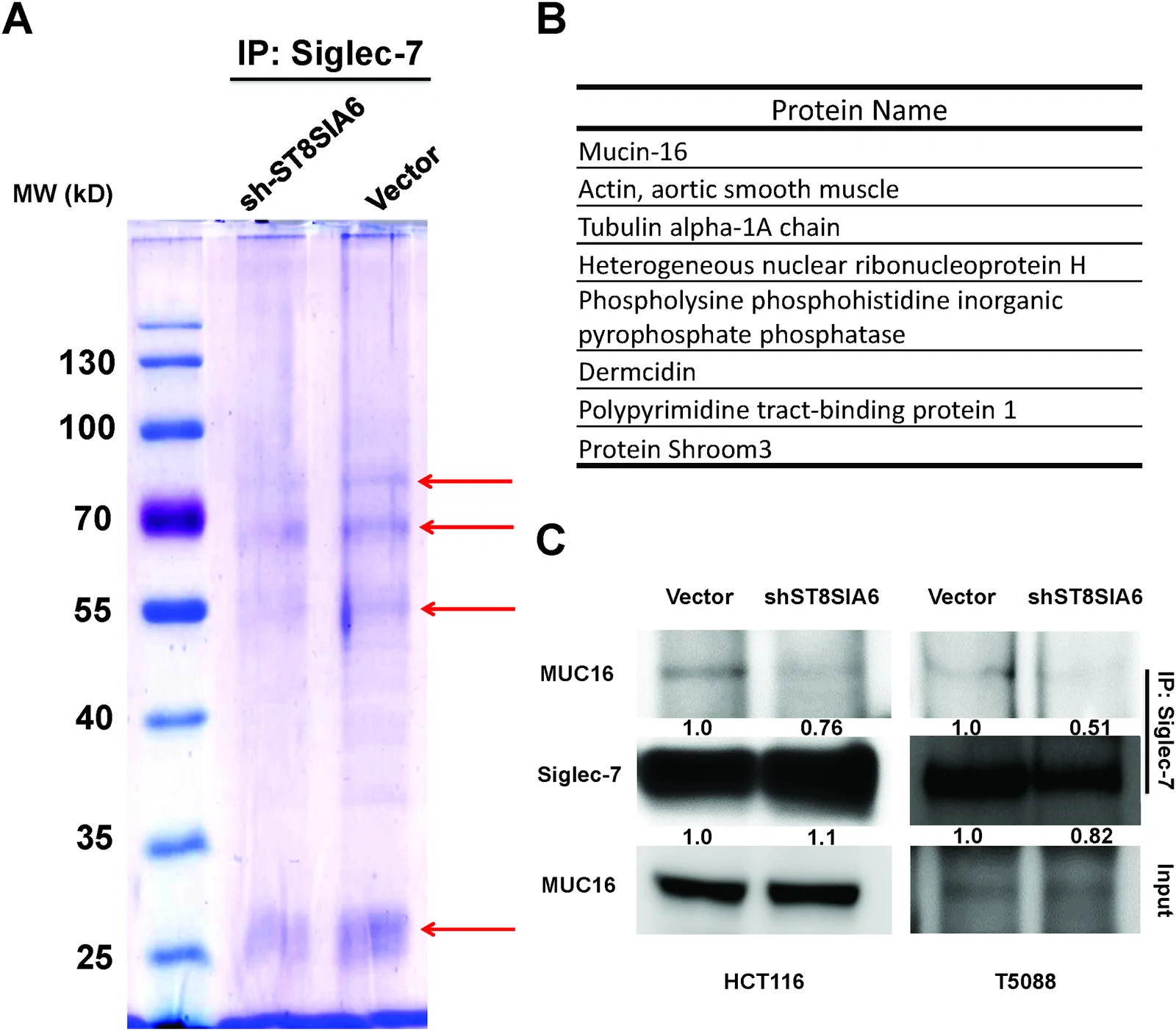
**ST8SIA6 knockdown reduces Siglec-7-reactive MUC16 in CRC cells. A.** Siglec-7-enriched proteins from vector-control and ST8SIA6-knockdown HCT116 cells were separated by SDS-PAGE and stained with Coomassie Brilliant Blue. The indicated gel regions from vector-control cells were excised, pooled, and collectively subjected to in-gel digestion followed by liquid chromatography-tandem mass spectrometry (LC-MS/MS). Because the excised gel regions were pooled before in-gel digestion, the LC-MS/MS output represents proteins identified collectively across these regions rather than proteins assigned to individual visible bands. Accordingly, the arrows indicate the excised gel regions and do not denote a one-to-one assignment between individual bands and specific proteins. **B.** Proteins identified by LC-MS/MS from the pooled Siglec-7-enriched gel regions. **C.** Siglec-7 pull-down followed by immunoblotting for MUC16 in HCT116 and T5088 cells. Representative Western blot images and densitometric quantification are shown; n = 3 biological replicates. Data are presented as mean ± SD. Statistical significance was determined using Student's t-test; p < 0.05.

To identify glycoproteins potentially affected by ST8SIA6 loss, we performed Siglec-7-based enrichment followed by protein identification. Because Siglec-7 preferentially recognizes disialylated glycan structures but does not provide complete structural specificity, this approach was used as a discovery strategy rather than definitive glycan mapping. By immunoprecipitation with Siglec-7, a lectin with preferential affinity for selected disialylated glycan structures, disialylated glycoproteins in HCT116 cells were pulled down and subjected to protein electrophoresis, followed by in-gel digestion for peptide identification (Fig. 3A). Several proteins were identified via liquid chromatography-mass spectrometry analysis (Fig. 3B). Among the enriched proteins, MUC16 was identified as one of the major Siglec-7-reactive membrane-associated glycoproteins. Total MUC16 expression was comparable between control and sh-ST8SIA6 cells, whereas Siglec-7-enriched MUC16 was reduced in sh-ST8SIA6 cells (Fig. 3C). These findings suggest that ST8SIA6 knockdown reduces the Siglec-7 reactivity of MUC16, although the precise MUC16 O-glycan structures remain unresolved. To further examine MUC16-associated glycans, immunopurified MUC16 was subjected to PNGase F release and permethylation-based *N*-glycan mass spectrometry. No clear difference in disialylated N-glycan compositions was detected between control and ST8SIA6-knockdown cells. However, because this analysis was restricted to released *N*-glycans and did not resolve sialic acid linkage, it does not address the extensively represented *O*-glycans of MUC16 or establish the presence or absence of α2,8-linked structures.

### 3.4 ST8SIA6 loss is associated with increased membrane localization of MUC16

**Fig. 4:**
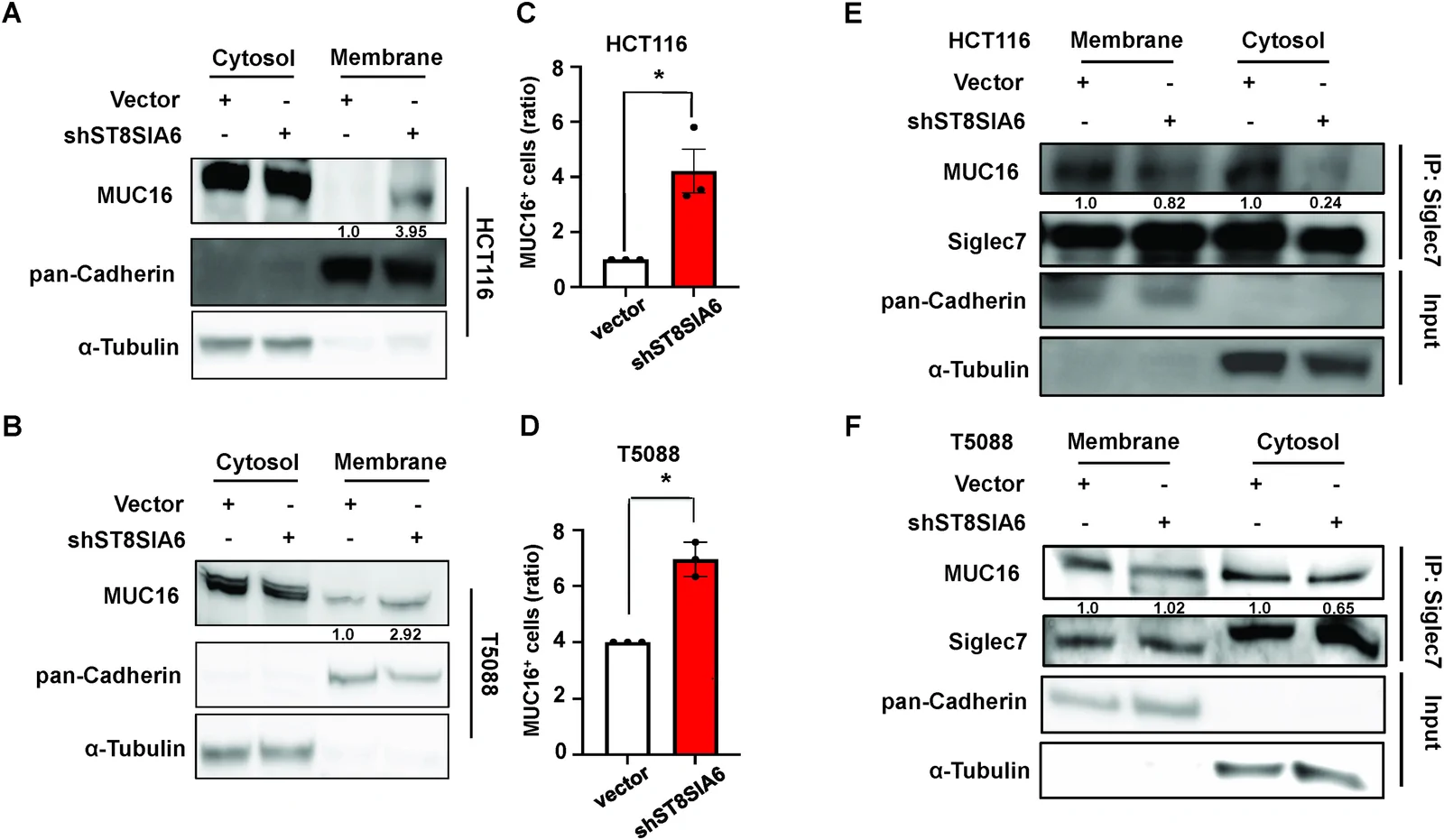
**Reduced ST8SIA6 expression in CRC cells enhances MUC16 membrane localization.** WB analysis of MUC16 expression in the membrane fraction of **A.** HCT116 and **B.** T5088 cells with vector or sh-*ST8SIA6* transfection, using pan-Cadherin and α-Tubulin as loading controls. Flow cytometry analysis of cell surface MUC16 in **C.** HCT116 and **D.** T5088 cells with vector or sh-*ST8SIA6.* Quantification data show the ratio of MUC16^+^ signals between the membrane and the cytosol. Immunoprecipitation of MUC16 by Siglec-7 in membrane and cytosol fractions in **E.** HCT116 and **F.** T5088 cells with vector or sh-*ST8SIA6* transfection. WB detected protein expression. Representative Western blot images and densitometric quantification are shown. n = 3 biological replicates. Data are mean ± SD. *p < 0.05 determined by Mann-Whitney *U* test.

Mucins are expressed in membrane-bound and secreted forms, reflecting their structure and function. Glycosylation can influence cell-surface glycoprotein localization [16]. Additionally, elevated MUC16 expression has been reported in multiple cancers and has been associated with disease progression [23,24,34,35]. To determine the localization of MUC16 in CRC cells, the cytosolic and membrane fractions of vector control and sh-ST8SIA6 cells were subjected to WB analysis of MUC16. Notably, MUC16 was elevated in the membrane fraction of sh-ST8SIA6 cells (Fig. 4A and B). Flow cytometry analysis also revealed higher percentages of surface-expressed MUC16 in sh-ST8SIA6 cells than in their respective vector control cells (Fig. 4C and D). Moreover, to assess Siglec-7 reactivity of membrane-associated MUC16, a pull-down assay using Siglec-7 from membrane and cytosol fractions showed reduced Siglec-7 reactivity of MUC16 in sh-ST8SIA6 cells compared with vector controls (Fig. 4E and F). These findings further indicate that ST8SIA6 loss is associated with reduced Siglec-7 reactivity of MUC16 and increased MUC16 abundance at the cell surface.

### 3.5 Increased membrane-associated MUC16 is associated with integrin β4-dependent Src/FAK signaling

**Fig. 5:**
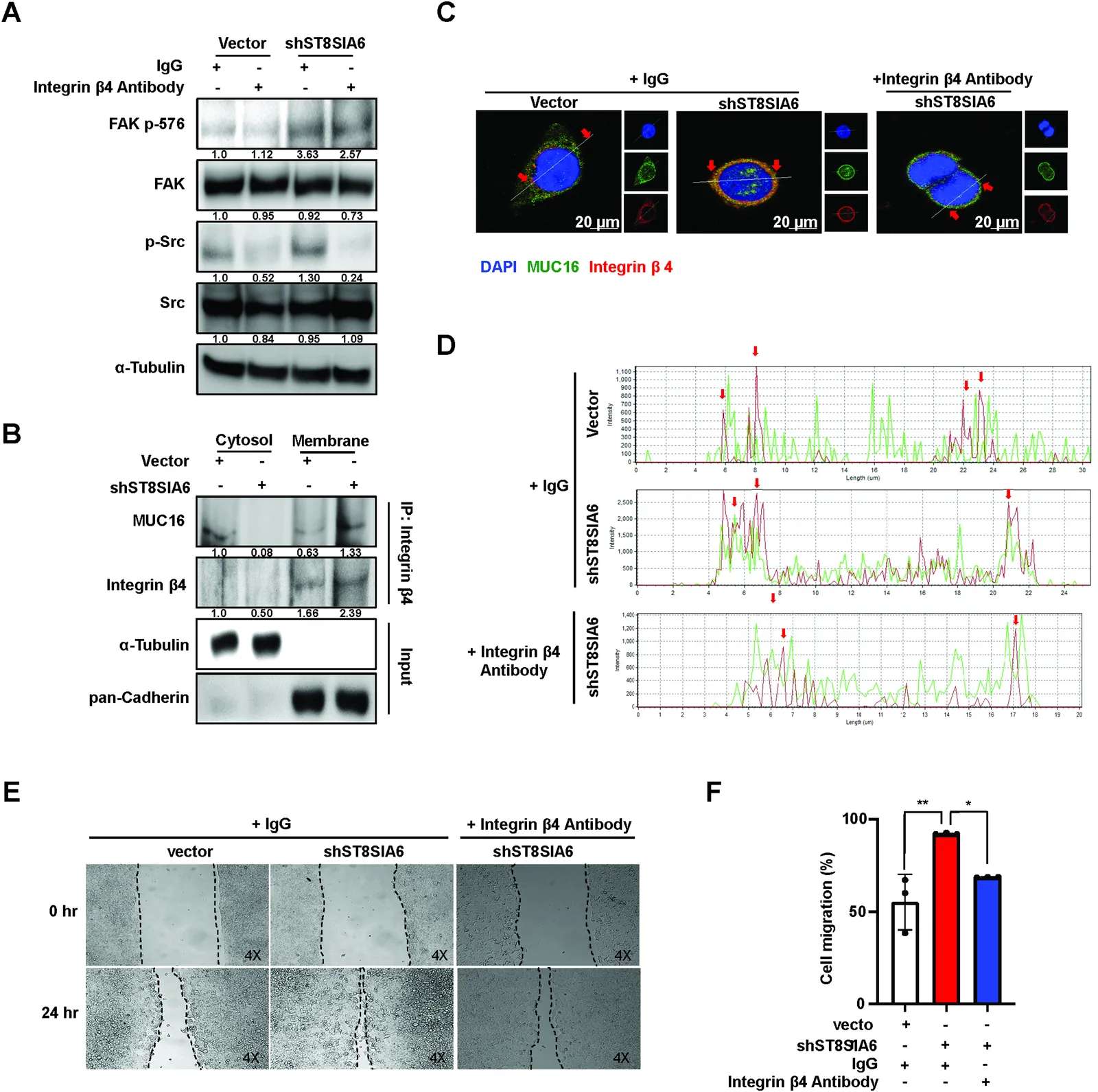
**Membrane-associated MUC16 exhibits increased association and colocalization with integrin β4 on the cell membrane, which is associated with HCT116 cell migration via the activation of FAK and Src**. **A.** HCT116 cells transfected with vector or sh-*ST8SIA6* were treated with IgG control or integrin β4 antibodies. WB analysis of phosphorylated FAK and Src in protein lysates after the treatment. **B.** Immunoprecipitation of MUC16 by integrin β4 antibody in membrane and cytosol fractions in HCT116 cells with vector or sh-*ST8SIA6* transfection. WB detected protein expression. **C.** The confocal microscopy images show colocalization of MUC16 and integrin β4 in HCT116 cells after vector or sh-ST8SIA6 transfection, with or without IgG control or integrin β4 antibody treatment. **D.** Line-scan analysis showing fluorescence intensity of MUC16 (green) and integrin β4 (red) along the indicated region. Red arrows indicate colocalization. **E.** The wound-healing assay was examined in HCT116 cells transfected with a vector or sh-ST8SIA6, with or without pretreatment with IgG control or integrin β4 antibodies. **F.** Representative light microscope images and quantification of the area of the migrated cells. Original magnification: ×40. Data are mean ± SD, *p < 0.05, **p < 0.01 determined by one-way ANOVA followed by Tukey's post hoc test. Representative Western blot images and densitometric quantification are shown. n = 3 biological replicates.

To investigate the underlying mechanism by which membrane MUC16 expression regulates CRC cell migration, we examined activation of Src/focal adhesion kinase (FAK) signaling, which promotes metastasis [36], in HCT116 (Fig. 5A-F) and T5088 (Fig. S1E–1J) cells. Our data revealed that silencing ST8SIA6 in HCT116 and T5088 cells increased phosphorylation of Src/FAK, and this effect was suppressed by a neutralizing integrin β4 antibody (Fig. 5A and Fig. S1E). This observation is consistent with previous studies demonstrating that Src and FAK act downstream of integrin β4 [26,27]. Furthermore, a stronger interaction between MUC16 and integrin β4 was observed in cells transfected with sh-*ST8SIA6* than in vector control cells (Fig. 5B and Fig. S1F). Immunofluorescence staining also revealed the colocalization of MUC16 with integrin β4 in sh-*ST8SIA6* cells (Fig. 5C and D, Figures S1G and 1H). This effect was not observed after treatment with an integrin-neutralizing antibody. Moreover, the migration of sh-*ST8SIA6* cells was diminished upon blockade of integrin β4 (Fig. 5E and F, Figures S1I and 1J). These findings associate reduced ST8SIA6 expression with increased MUC16-integrin β4 interaction, Src/FAK activation, and enhanced cell migration. Effects were modest in T5088 cells but consistent and stronger in HCT116.

### 3.6 Loss of ST8SIA6 is associated with increased lung colonization and MUC16-integrin β4 colocalization in vivo

**Fig. 6:**
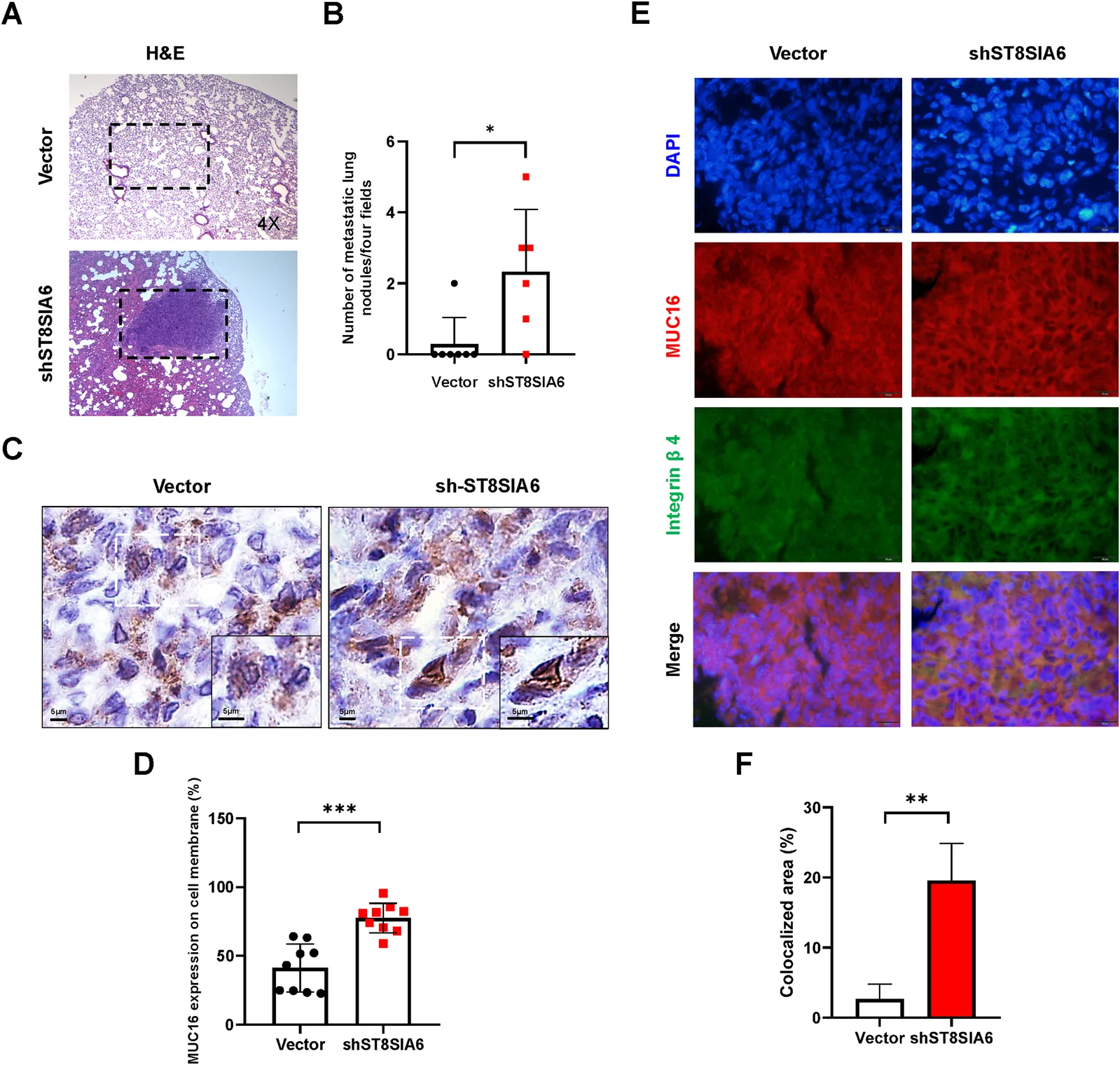
**ST8SIA6 reduction is associated with MUC16 membrane localization and MUC16-integrin β4 colocalization in xenografts. A.** H&E staining of lung tissue from mice with tail vein injection of vector and sh-*ST8SIA6* CRC cells. **B.** Quantification data show the number of nodules observed in the lung tissue. Data are mean ± SD. (n = 6 mice) *p < 0.05 determined by Mann-Whitney *U* test. **C.** IHC staining depicting expression of MUC16 in tumor tissues from mice subcutaneously injected with vector and sh-*ST8SIA6* CRC cells. **D.** Quantification data show the percentage of membrane MUC16 expression in these tumor tissues. Data are mean ± SD, ***p < 0.001 determined by Mann-Whitney *U* test. **E.** Immunofluorescence staining showing MUC16 (red) and integrin β4 (green) expression in tumor tissues from mice subcutaneously injected with vector and sh-*ST8SIA6* CRC cells (yellow color in merge image). **F.** Quantification data show the percentage of MUC16/integrin β4 colocalization (yellow color) in tumor tissues. Data are mean ± SD. *p < 0.05 determined by Mann-Whitney *U* test.

We next validated these findings in NOD/SCID mice, which were injected via the tail vein with either vector control or sh-*ST8SIA6* HCT116 cells. Six weeks post-injection, lung tissues were harvested and subjected to H&E staining. The number of lung nodules was significantly increased in the mice injected with sh-*ST8SIA6* CRC cells (Fig. 6A and B), indicating that ST8SIA6 deficiency is associated with increased lung tumor colonization. Moreover, to investigate MUC16 translocation to the cell membrane and its colocalization with integrin β4 in vivo, HCT116 cells with either the control vector or sh-*ST8SIA6* were subcutaneously injected into NOD/SCID mice. Increased membrane localization of MUC16 was detected in tumor tissues from the sh-*ST8SIA6* group (Fig. 6C and D). Immunofluorescence also revealed notable colocalization between MUC16 and integrin β4 in the sh-*ST8SIA6* group compared with the control group (Fig. 6E and F). These results show that ST8SIA6 loss is associated with increased membrane localization of MUC16 and greater MUC16-integrin β4 colocalization in subcutaneous tumors. Together with the tail-vein findings, these observations are consistent with an aggressive in vivo phenotype.

### 3.7 Elevated MUC16 expression and MUC16-integrin β4 colocalization are observed in advanced human colorectal cancer

**Fig. 7:**
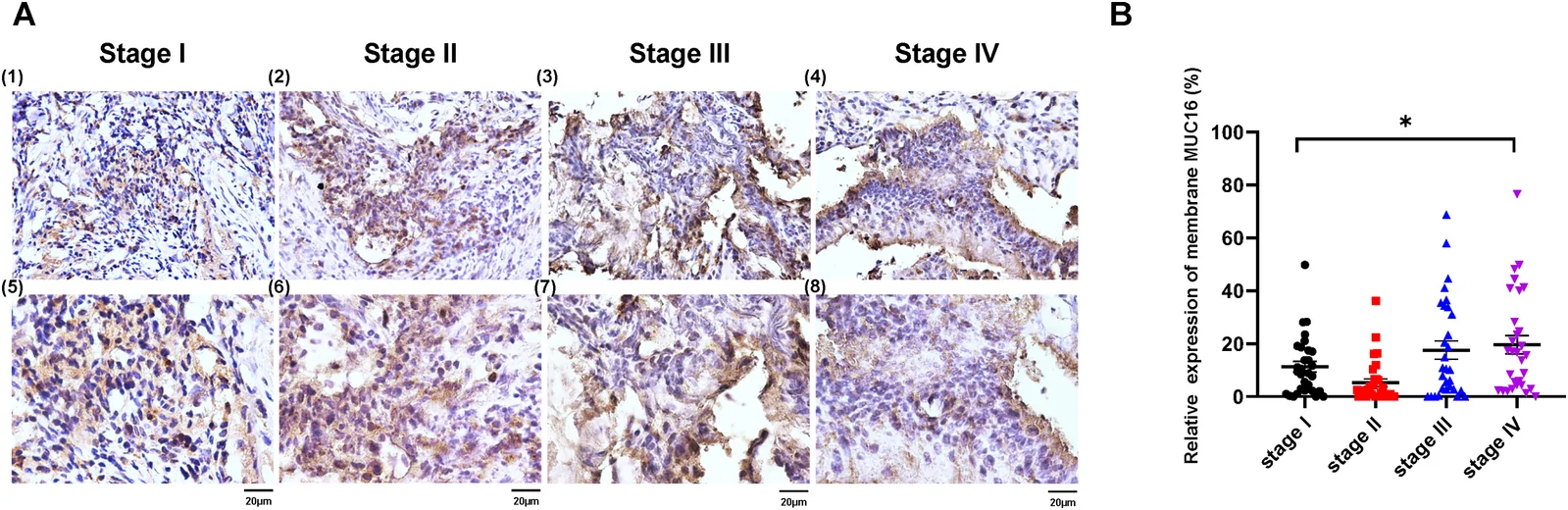
**MUC16 expression is elevated in advanced colorectal cancer patients.** All the tissues were from the Bio-Bank of the Medical Research Department, E-DA Hospital. **A.** Representative IHC images of MUC16 in mucosal tissues from CRC patients. The lower row (5)-(8) shows enlarged images of the frame area corresponding to the upper row (1)-(4). Scale bars: as indicated in figure. B. The expression of MUC16 on the membrane in each stage was quantified using 40 fields. Original magnification: ×40. Data are mean ± SEM. *p < 0.05 determined by Kruskal-Wallis test followed by Dunn's multiple-comparison test.

**Fig. 8:**
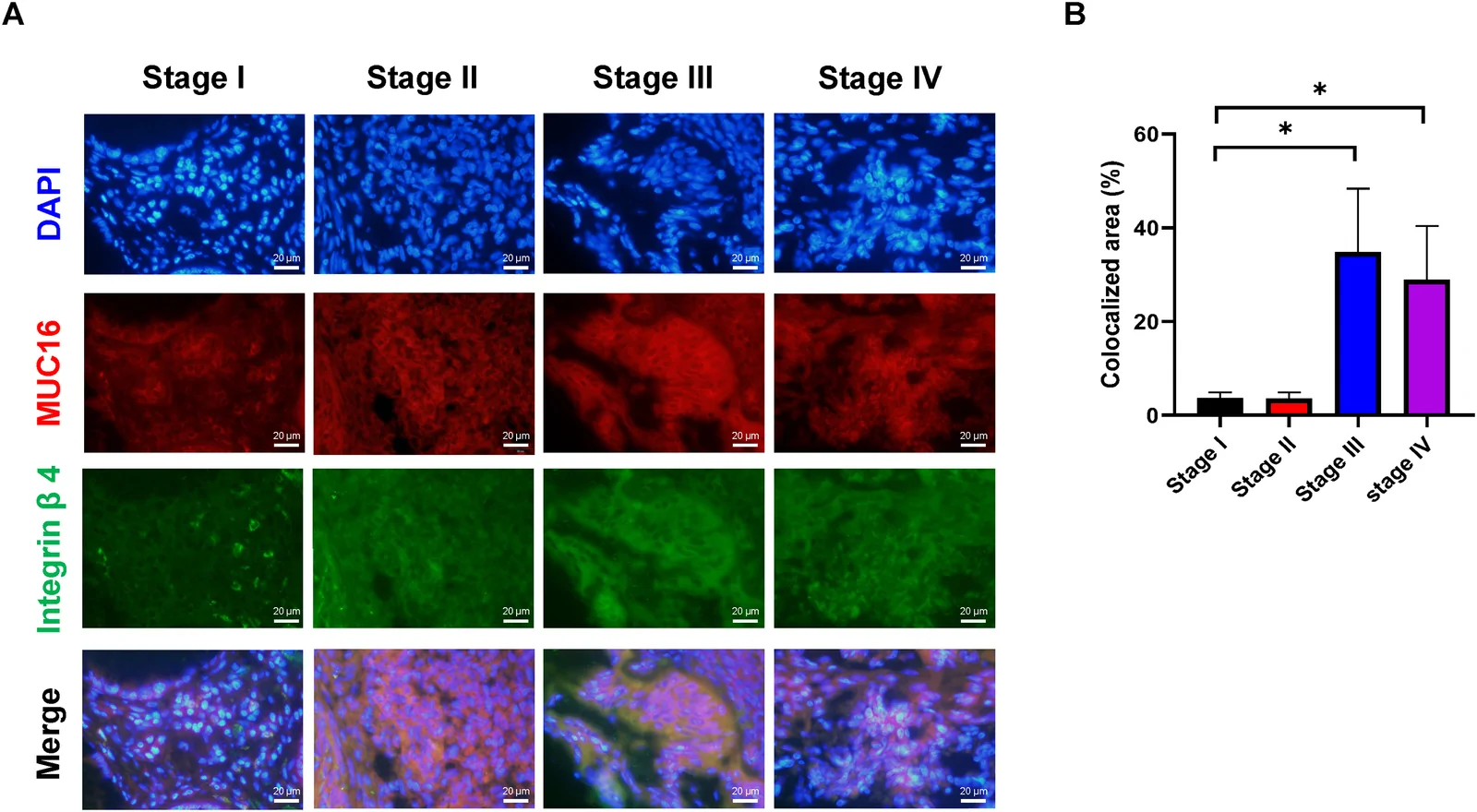
**MUC16 colocalizes with integrin β4 in patients with colorectal cancer.** All tissues were obtained from the Bio-Bank of the Medical Research Department at E-DA Hospital. Representative immunofluorescence images showing colocalization of MUC16 (red) and integrin β4 (green) in mucosal tissues of colorectal cancer patients across Stages I-IV, with DAPI (blue) staining nuclei. Scale bar: 20 μm. Three fields per patient were analyzed. Original magnification: ×40. **B.** Quantification of MUC16/integrin β4 colocalized area (%) across stages, calculated from 3 fields per patient. Data are mean ± SEM, **p < 0.01 determined by Kruskal-Wallis test followed by Dunn's multiple-comparison test.

Next, we assessed MUC16 expression in human CRC tissues. Through IHC staining, a significant increase in MUC16 expression was observed in stage IV patients (Fig. 7A and B). Immunofluorescence staining was performed to assess colocalization of MUC16 and integrin β4 in human CRC tissues. The colocalization of MUC16 and integrin β4 within mucosal and tumor regions was detected and was greater in advanced-stage patients than in stage I patients (Fig. 8A and B). These results indicate that membrane-associated MUC16 and its colocalization with integrin β4 are associated with advanced CRC, suggesting activation of this signaling axis in advanced CRC. Consistent with GEPIA analyses showing elevated MUC16 expression in CRC tumors (Fig. S4), our tissue microarray similarly indicates stage-associated elevation of MUC16. Our IHC detects ST8SIA6/MUC16 in subsets of CRC tumors, potentially due to differences in antibodies or tissue processing.

## 4 Discussion

In this study, we demonstrate that reduced ST8SIA6 expression is associated with enhanced migration and invasion of colorectal cancer cells and increased experimental lung colonization following tail-vein injection. Collectively, our findings support a model in which loss of ST8SIA6 alters Siglec-7 reactivity of MUC16, increases membrane localization of MUC16, and enhances integrin β4-dependent Src/FAK signaling.

Previous studies have demonstrated that ST8SIA6 participates in multiple biological processes, including immune regulation through Siglec-mediated signaling and modulation of glycoprotein function [[16], [17], [18]].

The biological consequences of ST8SIA6 appear to be highly context-dependent, with tumor-promoting effects reported in some experimental settings [11,17,18], whereas our previous CRC analyses associated reduced ST8SIA6 expression with more advanced disease [12,19]. Consistent with our previous bioinformatic analyses, we observed decreased ST8SIA6 expression in advanced-stage CRC tissues. These findings extend earlier observations by suggesting that reduced ST8SIA6 expression may influence tumor cell behavior through alterations in cell-surface glycoprotein signaling rather than exclusively through immune-regulatory mechanisms.

A principal finding of this study is the identification of MUC16 as a prominent Siglec-7-reactive glycoprotein associated with ST8SIA6. MUC16 is widely recognized as a tumor-associated mucin and has been implicated in tumor progression through regulation of cell adhesion, migration, and intracellular signaling. However, the influence of glycosylation on MUC16 localization in CRC has not been well characterized. Our results indicate that ST8SIA6 knockdown reduces the Siglec-7 reactivity of MUC16 while increasing its abundance at the plasma membrane. These observations suggest that ST8SIA6-associated glycosylation may contribute to the regulation of MUC16 membrane localization. Because MUC16 is an exceptionally large and heavily O-glycosylated mucin, definitive characterization of its glycan structures remains technically challenging. Accordingly, our data should be interpreted as demonstrating altered Siglec-7 reactivity rather than direct structural evidence of α2,8-linked sialylation on MUC16.

The mechanism by which altered glycosylation influences MUC16 membrane localization remains to be determined. Glycosylation has been reported to regulate protein trafficking, membrane stability, and interactions with extracellular binding partners. In the present study, increased membrane-associated MUC16 was consistently accompanied by enhanced association with integrin β4 and increased phosphorylation of Src and FAK. Furthermore, neutralization of integrin β4 attenuated Src/FAK activation and reduced cell migration, supporting a functional role for integrin β4 signaling downstream of ST8SIA6 loss. Although additional studies are required to establish the precise molecular mechanism, these findings support a model that may contribute to increased membrane localization of MUC16, thereby favoring its association with integrin β4.

The biological relevance of this signaling axis is supported by both experimental models and clinical specimens. ST8SIA6-deficient CRC cells exhibited increased lung tumor colonization following tail-vein injection, together with increased membrane localization of MUC16 and enhanced MUC16-integrin β4 colocalization in xenograft tumors. Likewise, advanced-stage human CRC tissues displayed increased membrane-associated MUC16 and greater colocalization with integrin β4 than early-stage tumors. Although the tail-vein model evaluates experimental metastatic colonization rather than the complete metastatic cascade, these observations are consistent with the in vitro migration and invasion data and collectively support the association between reduced ST8SIA6 expression and aggressive CRC phenotypes.

Several limitations of this study should be acknowledged. First, although Siglec-7 enrichment combined with proteomic analysis identified MUC16 as a prominent ST8SIA6-associated glycoprotein, the precise glycan structures responsible for Siglec-7 binding were not determined. An exploratory released *N*-glycan analysis did not identify a clear difference in disialylated *N*-glycan compositions; however, the analysis was restricted to *N*-glycans and was not linkage-specific, and therefore does not resolve the relevant MUC16 *O*-glycan structures. Future glycoproteomic analyses will be required to define these structures directly. Second, only a single shRNA was used for ST8SIA6 knockdown, and rescue experiments or independent genetic perturbation were not performed. Therefore, causal relationships should be interpreted with appropriate caution. Third, although integrin β4 blockade reduced Src/FAK activation and cell migration, direct genetic validation of MUC16 function was beyond the scope of the present study. Finally, dedicated proliferation and viability controls were not included; therefore, indirect effects on cell growth cannot be completely excluded. These limitations should be considered when interpreting the proposed mechanism.

Despite these limitations, our findings identify a previously unrecognized association between ST8SIA6 expression, MUC16 membrane localization, and integrin β4 signaling in colorectal cancer. The observed reduction of ST8SIA6 in advanced tumors suggests that this pathway may have potential value for stratifying aggressive disease. In addition, the downstream MUC16-integrin β4-Src/FAK signaling axis may represent a candidate therapeutic target for tumors with reduced ST8SIA6 expression. Further studies using orthotopic models, complementary genetic approaches, and detailed glycoproteomic analyses will help clarify the molecular basis and clinical significance of this pathway.

In conclusion, our study demonstrates that loss of ST8SIA6 is associated with altered Siglec-7 reactivity of MUC16, increased membrane localization of MUC16, enhanced integrin β4-dependent Src/FAK signaling, and aggressive cellular phenotypes in colorectal cancer. These findings provide new insight into how alterations in glycosylation may influence tumor-associated signaling networks and establish a foundation for future investigations of ST8SIA6-regulated glycoprotein biology in CRC.

## Author contributions

Conceptualization: Pei-Chun Shih, Chuan-Fa Chang.

Methodology: Pei-Chun Shih, Yung-Kuo Lee.

Investigation: Pei-Chun Shih, Ching-Cheng Hsu, Tsung-Hsien Lin, Ngoc Uyen Nhi Nguyen.

Formal analysis: Pei-Chun Shih, Yung-Kuo Lee.

Resources: Hsin-Pao Chen.

Writing - original draft: Pei-Chun Shih.

Writing - review and editing: Wei-Ling Lin, Chuan-Fa Chang.

Supervision: Wei-Ling Lin, Chuan-Fa Chang.

All authors reviewed and approved the contribution statement.

## Ethics declaration

Written informed consent to take part in the study and to publish the article has been obtained from all participants or their legal representatives. The privacy rights of participants have been observed.

This study included organ or tissue donors. This study includes human biological material and consent was obtained by donors, or their next of kin or legal representatives, for use in this study and for publication of the article. The samples used in this research were not sourced from executed prisoners or prisoners of conscience.

This study was performed in compliance with relevant laws, regulatory frameworks and guidelines where the research took place. This study was approved by the nstitutional Review Board of E-DA Hospital. (Approval No. EMRP22107 N)

This study was conducted in accordance with the ARRIVE (Animal Research: Reporting of In Vivo Experiments) guidelines. This study was approved by the Laboratory Animal Center of National Cheng Kung University Medical College. (Approval No. 111208)

## Ethics approval and consent to participate

This study was approved by the Institutional Review Board of E-DA Hospital. IRB No: EMRP22107 N.

## Availability of data and materials

The mass spectrometry proteomics data have been deposited to the ProteomeXchange Consortium via the PRIDE partner repository [37] with the dataset identifier PXD077963. The authors confirm that the data supporting the findings of this study are available in the article and its Supplementary Information.

## Funding

This work was supported by grants from the 10.13039/100020595National Science and Technology Council, R.O.C, Taiwan (NSTC 115-2321-B-006-022, 114-2327-B-006-004, 113-2327-B-006-003, 113-2320-B-006-031, 111-2327-B-006-010, and 108-2314-B-650-010) and E-DA Hospital (NCKUEDA 10715).

## Declaration of competing interests

The authors declare that they have no known competing financial interests or personal relationships that could have appeared to influence the work reported in this paper.

## Data Availability

Proteomics data are available in PRIDE (PXD077963). All other supporting data are included in the article and the Supplementary Information.
