# Supplementary material for: Loss of ST8SIA6 is associated with enhanced MUC16-Integrin β4 signaling and aggressive phenotypes in colorectal cancer
Source: Biochem Biophys Rep. 2026 Aug 25;47:102757. doi: 10.1016/j.bbrep.2026.102757 (PMC13543091; doi:10.1016/j.bbrep.2026.102757)
Supplement: Supplementary file 1 [file mmc1.pdf]

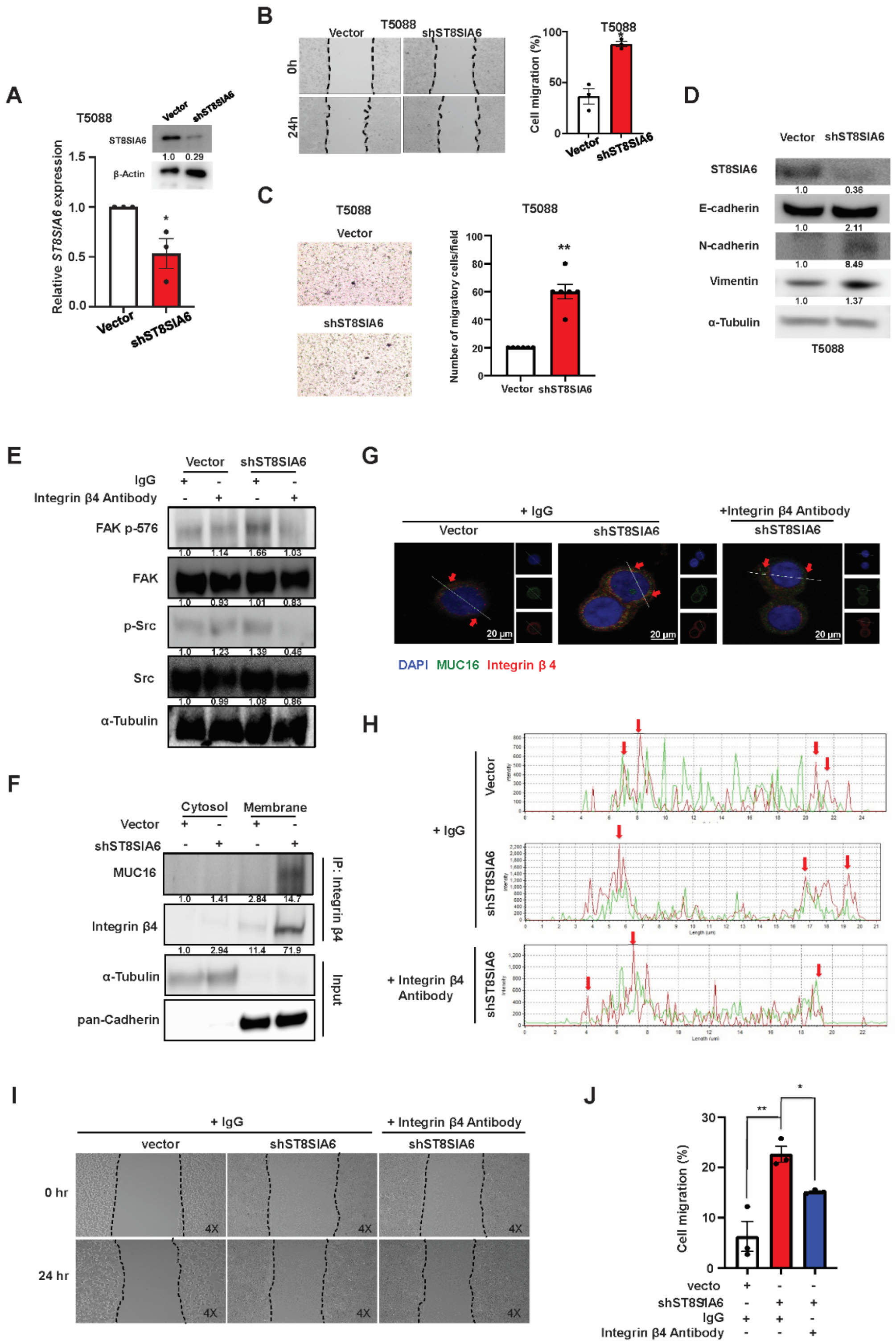


**Supp. Fig 1. A.** RT-qPCR and Western blot analysis of ST8SIA6 expression in T5088 cells transfected with control vector or sh-ST8SIA6, using β-actin as a loading control. Data are mean ± SD from three biological replicates. **p < 0.01, Student's t-

test. **B.** Wound-healing assay in T5088 cells transfected with vector or sh-ST8SIA6. Representative light-microscope images at 0 h and 24 h, and quantification of the migrated area are shown. Original magnification: ×4. Data are mean ± SD from three biological replicates. *$p < 0.05$, Student's t-test. **C.** Transwell migration assay in T5088 cells transfected with vector or sh-ST8SIA6. Representative crystal violet staining images and quantification of migrated cells are shown. Original magnification: ×100. Data are mean ± SD from three biological replicates. **$p < 0.01$, Student's t-test. **D.** Representative Western blot images and densitometric quantification of EMT-like markers, including E-cadherin, N-cadherin, and vimentin, in T5088 cells transfected with vector or sh-ST8SIA6, using α-tubulin as a loading control. $n = 3$ biological replicates. **E.** T5088 cells transfected with vector or sh-ST8SIA6 were treated with IgG control or integrin β4 antibody. Western blot analysis and densitometric quantification of phosphorylated FAK and Src are shown. $n = 3$ biological replicates. For multi-group comparisons, data were analyzed by one-way ANOVA followed by Tukey's post hoc test. **F.** Immunoprecipitation of MUC16 by integrin β4 antibody in membrane and cytosol fractions from T5088 cells transfected with vector or sh-ST8SIA6. Western blot analysis and densitometric quantification are shown. $n = 3$ biological replicates. **G.** Confocal microscopy images showing colocalization of MUC16 and integrin β4 in T5088 cells transfected with vector or sh-

ST8SIA6 after treatment with IgG control or integrin β4 antibody. **H.** Line-scan analysis showing fluorescence intensity of MUC16 and integrin β4 along the indicated region. Red arrows indicate colocalization. **I.** Wound-healing assay in T5088 cells transfected with vector or sh-ST8SIA6, with or without pretreatment with IgG control or integrin β4 antibody. **J.** Representative light-microscope images and quantification of the migrated area. Original magnification: ×40. Data are mean ± SD from three biological replicates. For multi-group comparisons, data were analyzed by one-way ANOVA followed by Tukey's post hoc test. *$p < 0.05$, **$p < 0.01$.

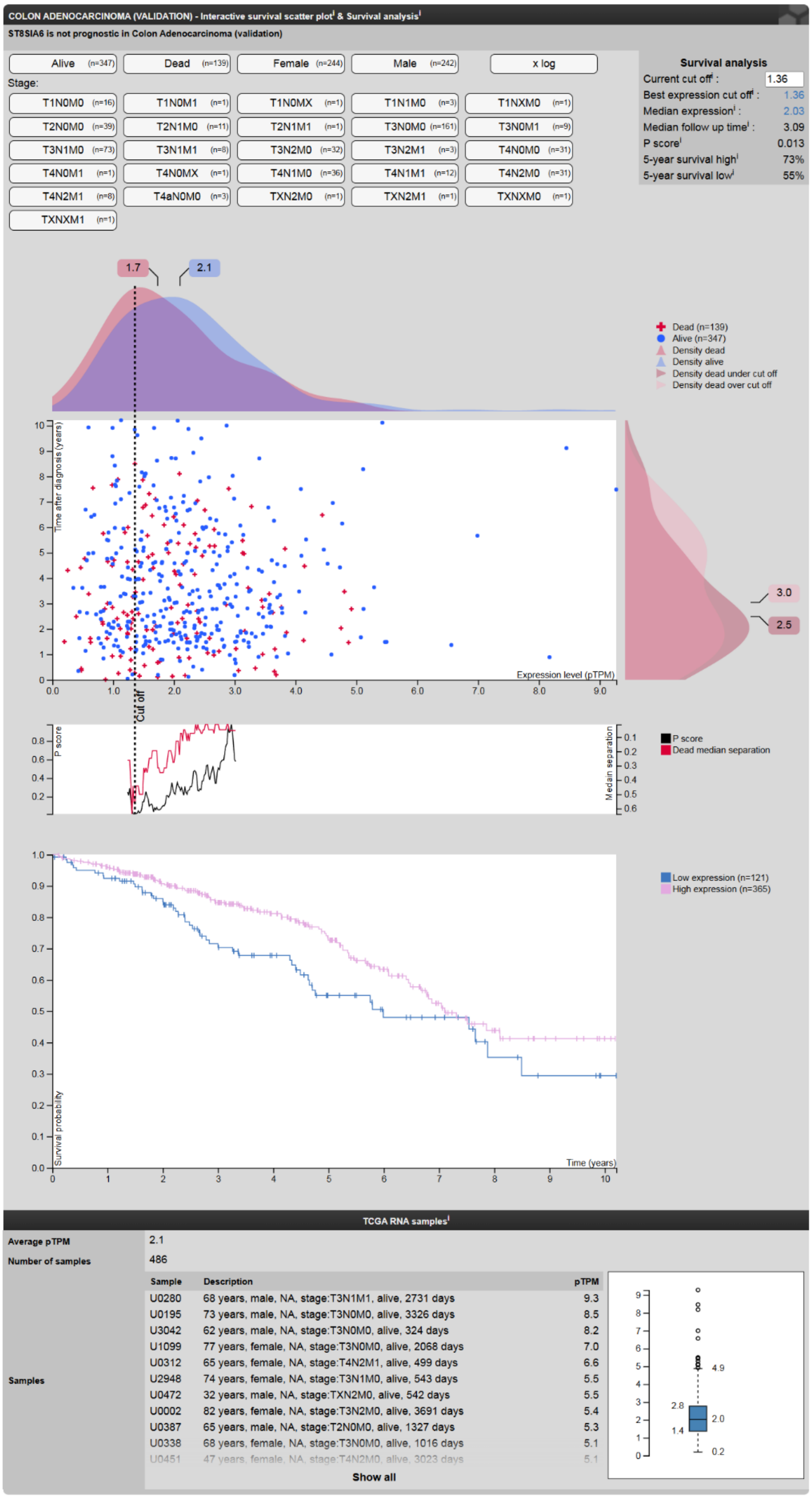


**Supp. Fig 2.** Survival analysis of ST8SIA6 expression in colon adenocarcinoma from HPA.

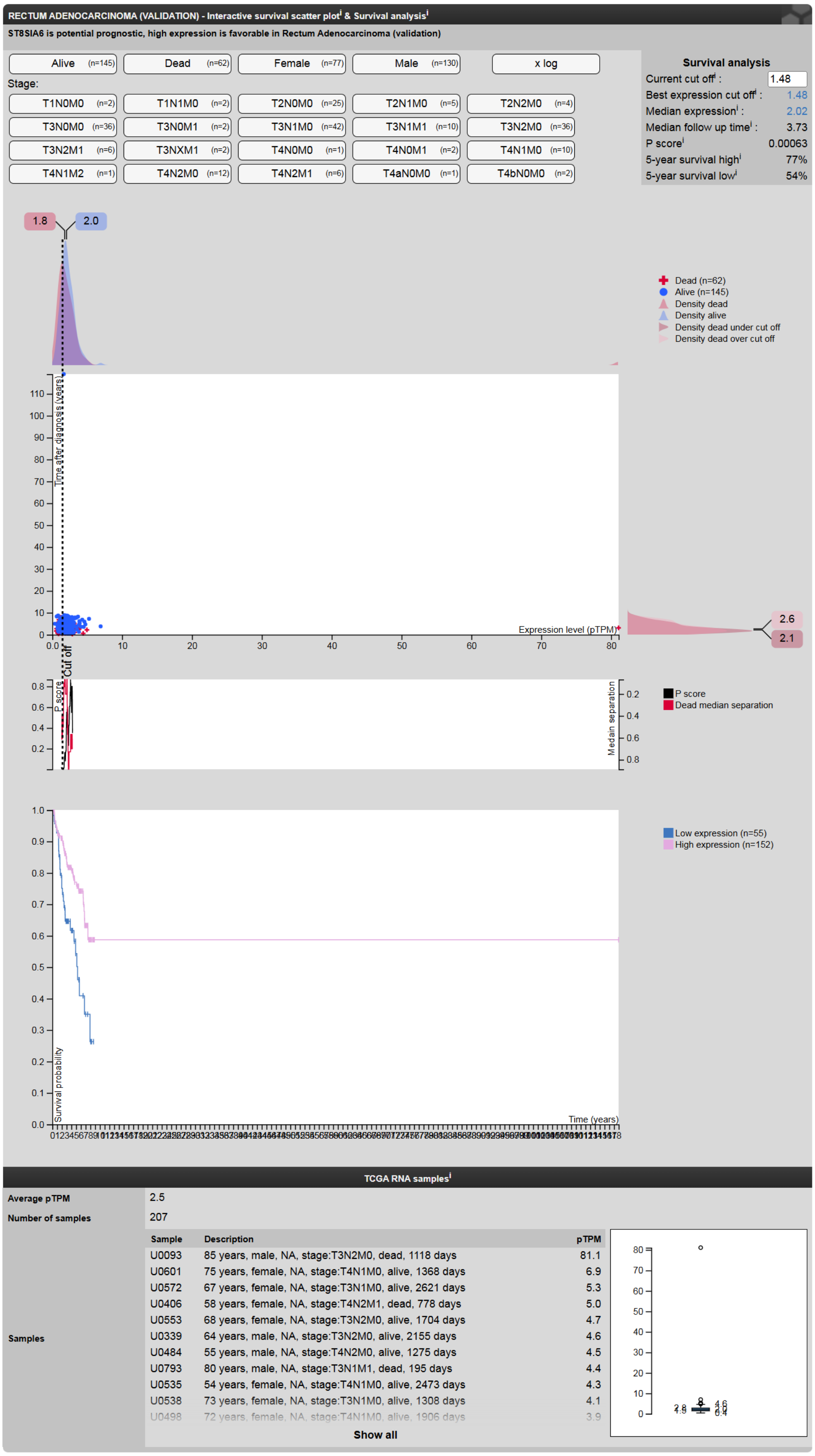


**Supp. Fig 3.** Survival analysis of ST8SIA6 expression in rectum adenocarcinoma from HPA.

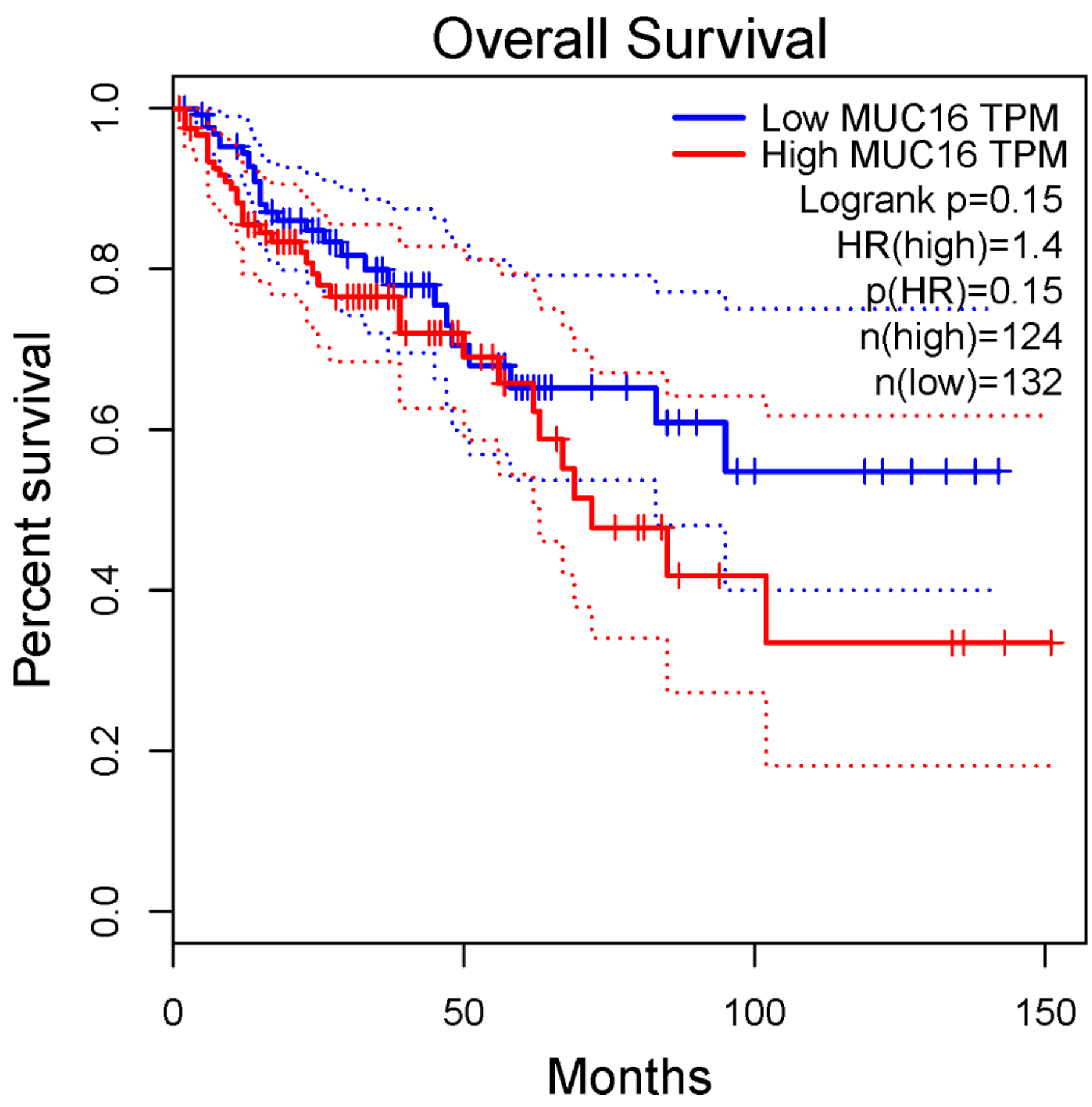


**Supp. Fig 4.** Survival analysis of MUC16 expression in colon adenocarcinoma from GEPIA.
