# Supplementary material for: Loss of ST8SIA6 is associated with enhanced MUC16-Integrin β4 signaling and aggressive phenotypes in colorectal cancer
Source: Biochem Biophys Rep. 2026 Aug 25;47:102757. doi: 10.1016/j.bbrep.2026.102757 (PMC13543091; doi:10.1016/j.bbrep.2026.102757)
Supplement: Supplementary file 2 [file mmc2.pdf]

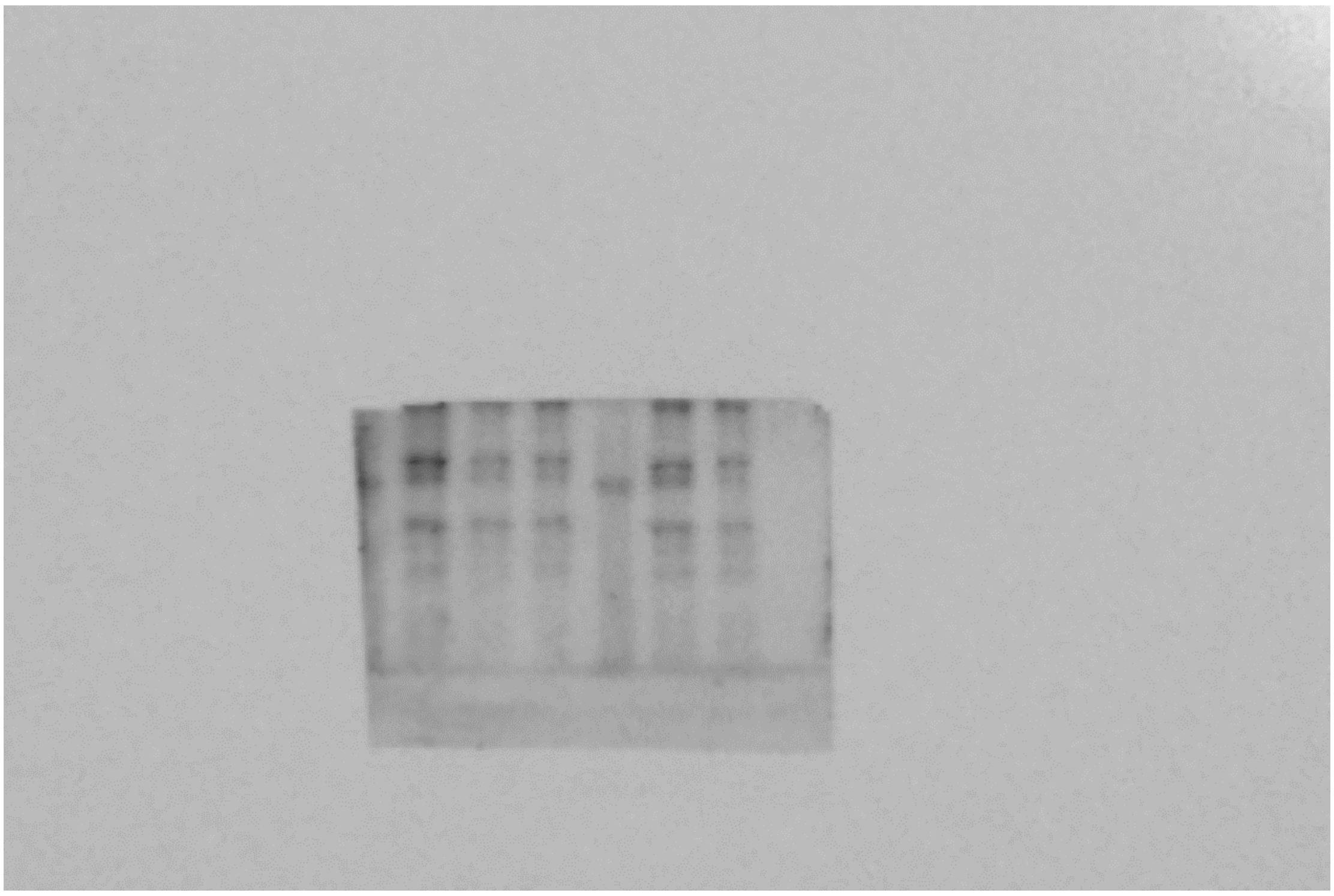

Fig-2A ST8SIA6

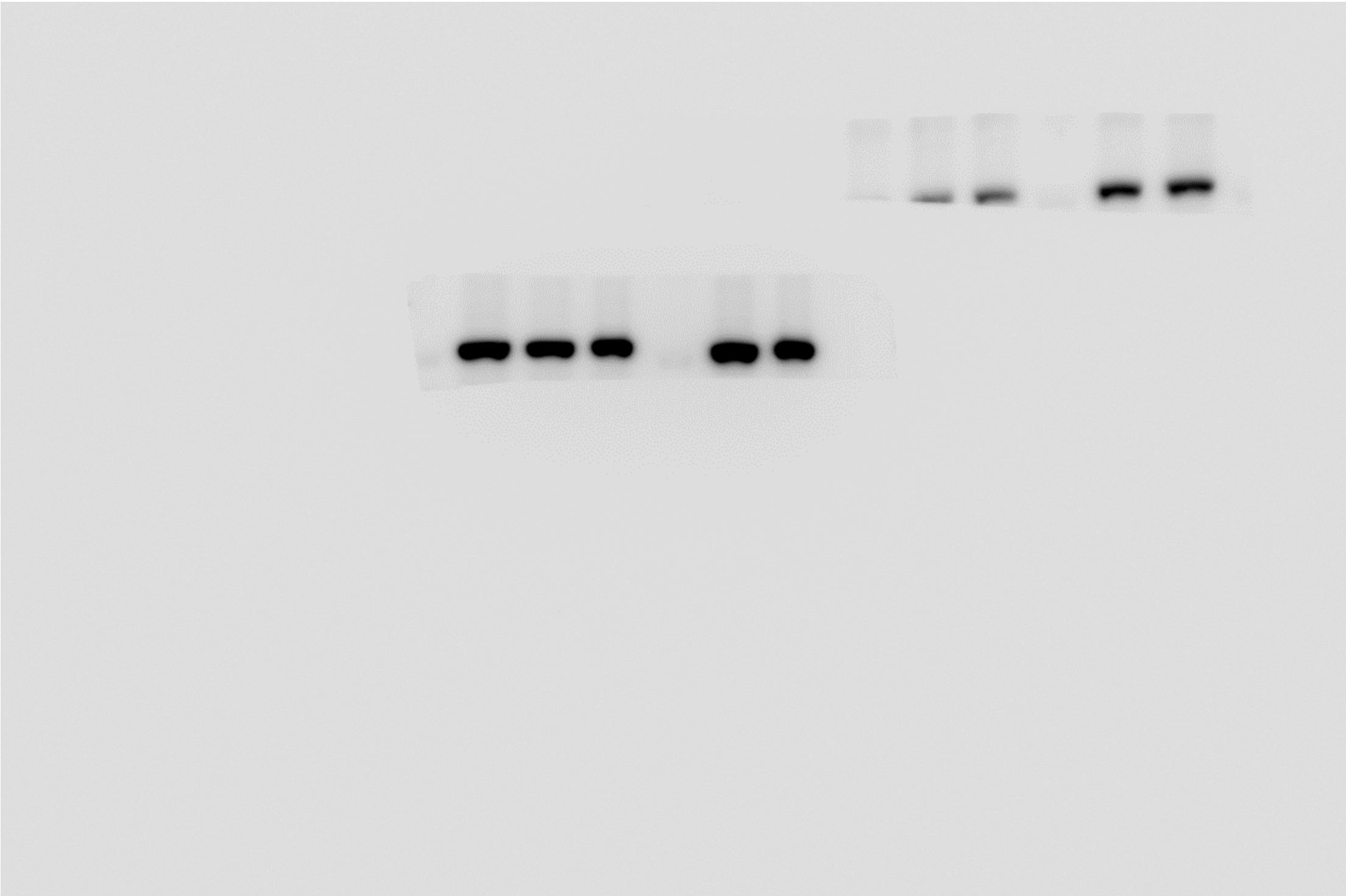

Fig-2A Tubulin

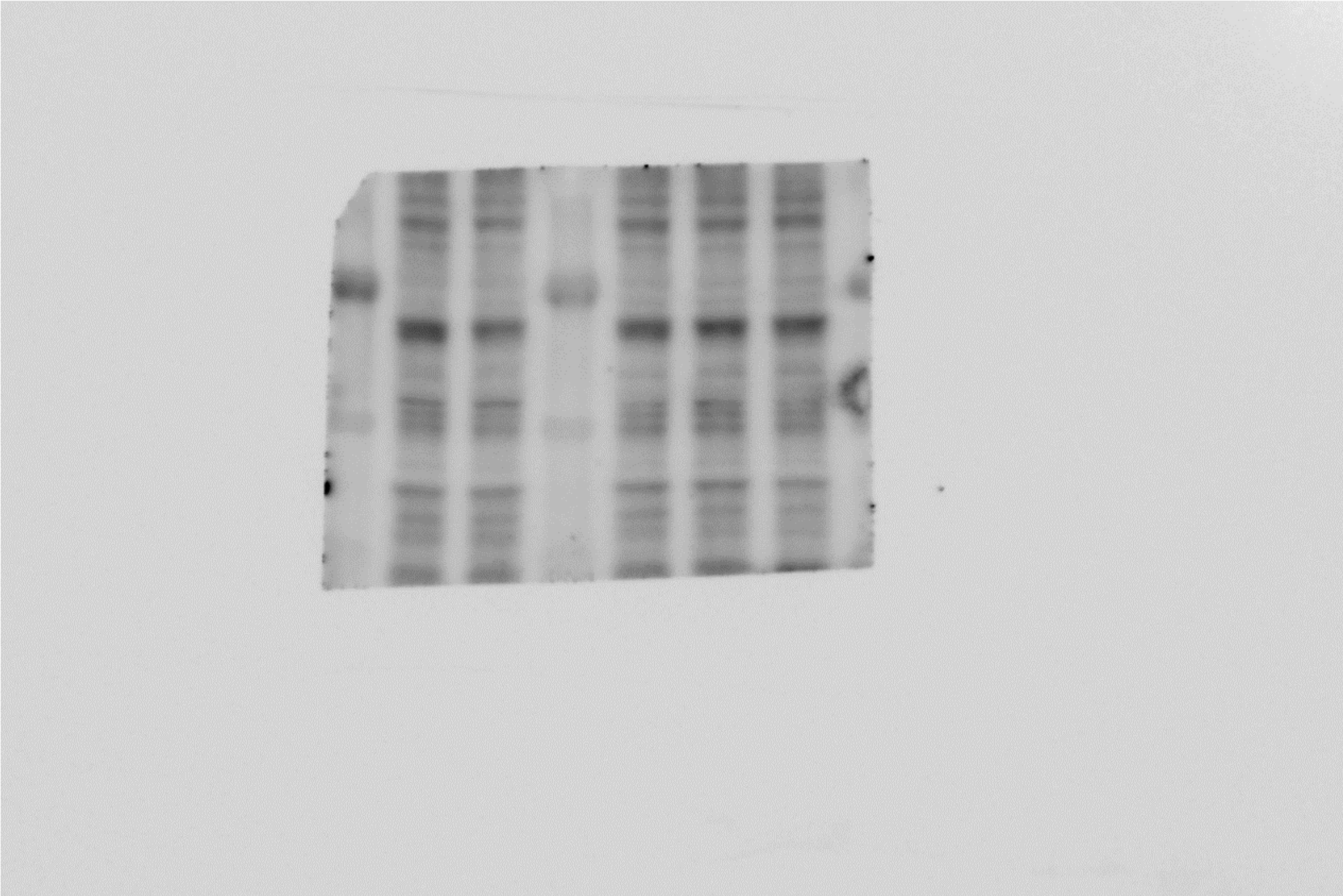

Fig-2D ST8SIA6

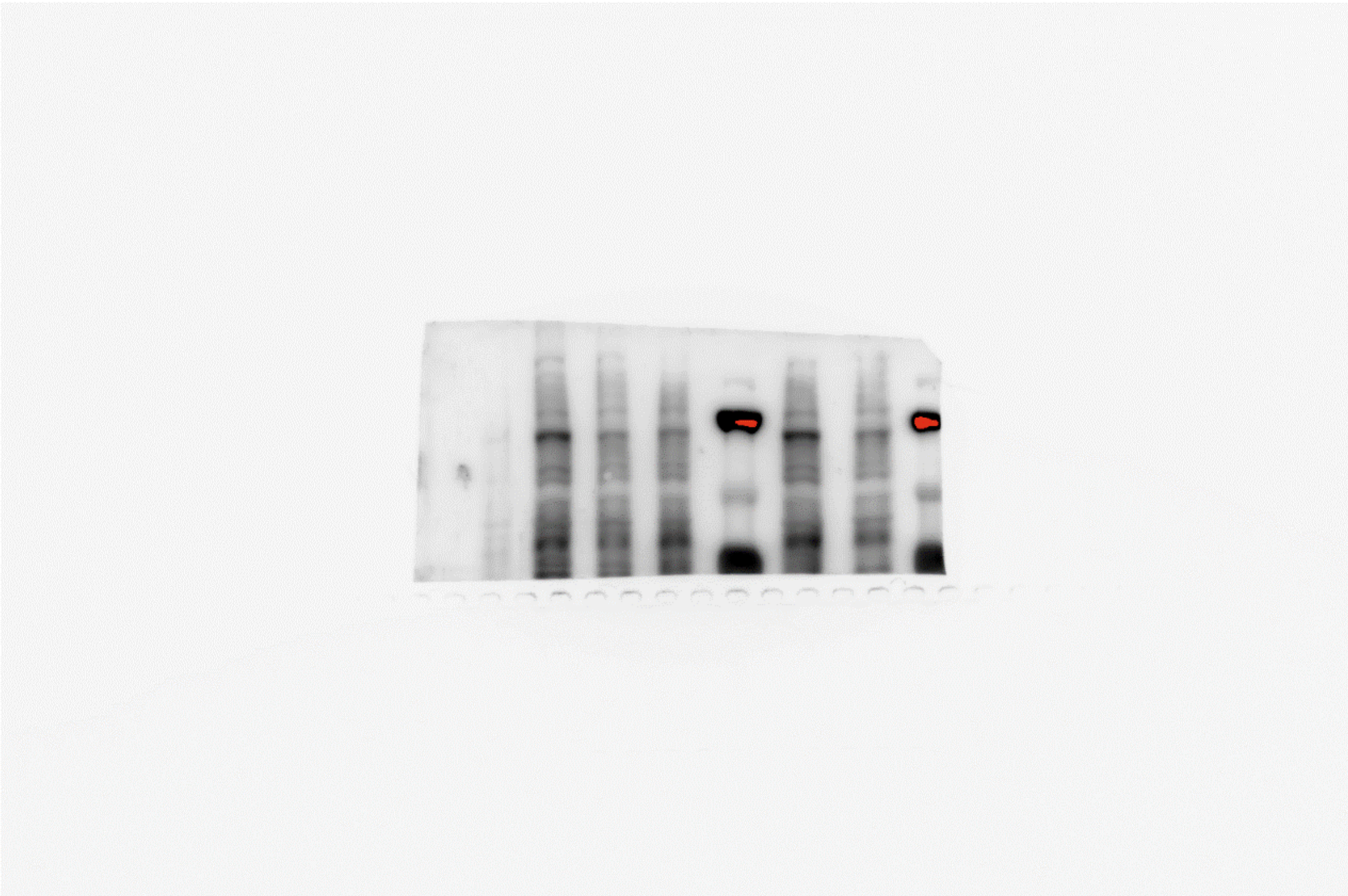

Fig-2D E-cadherin

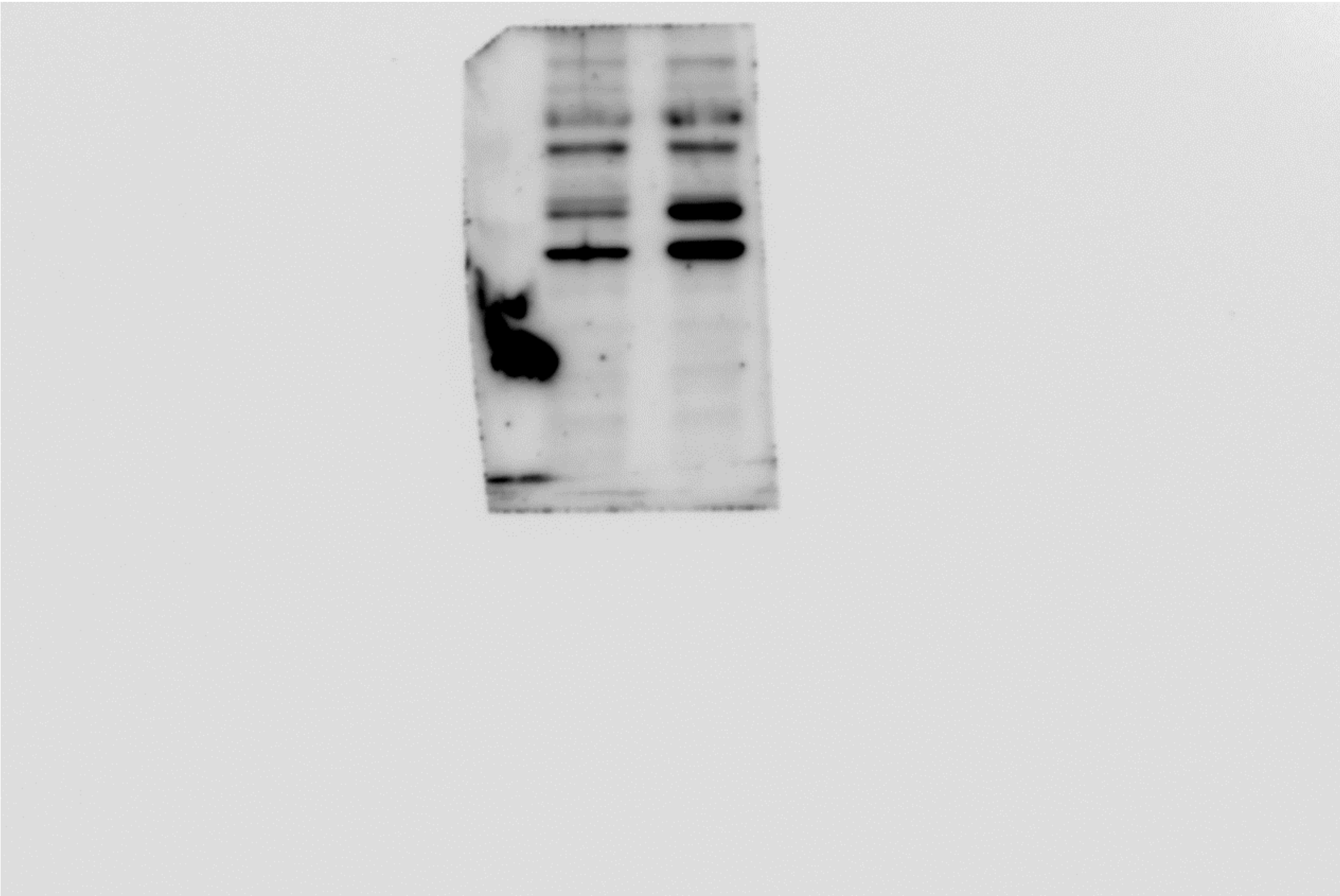

Fig-2D Vimentin

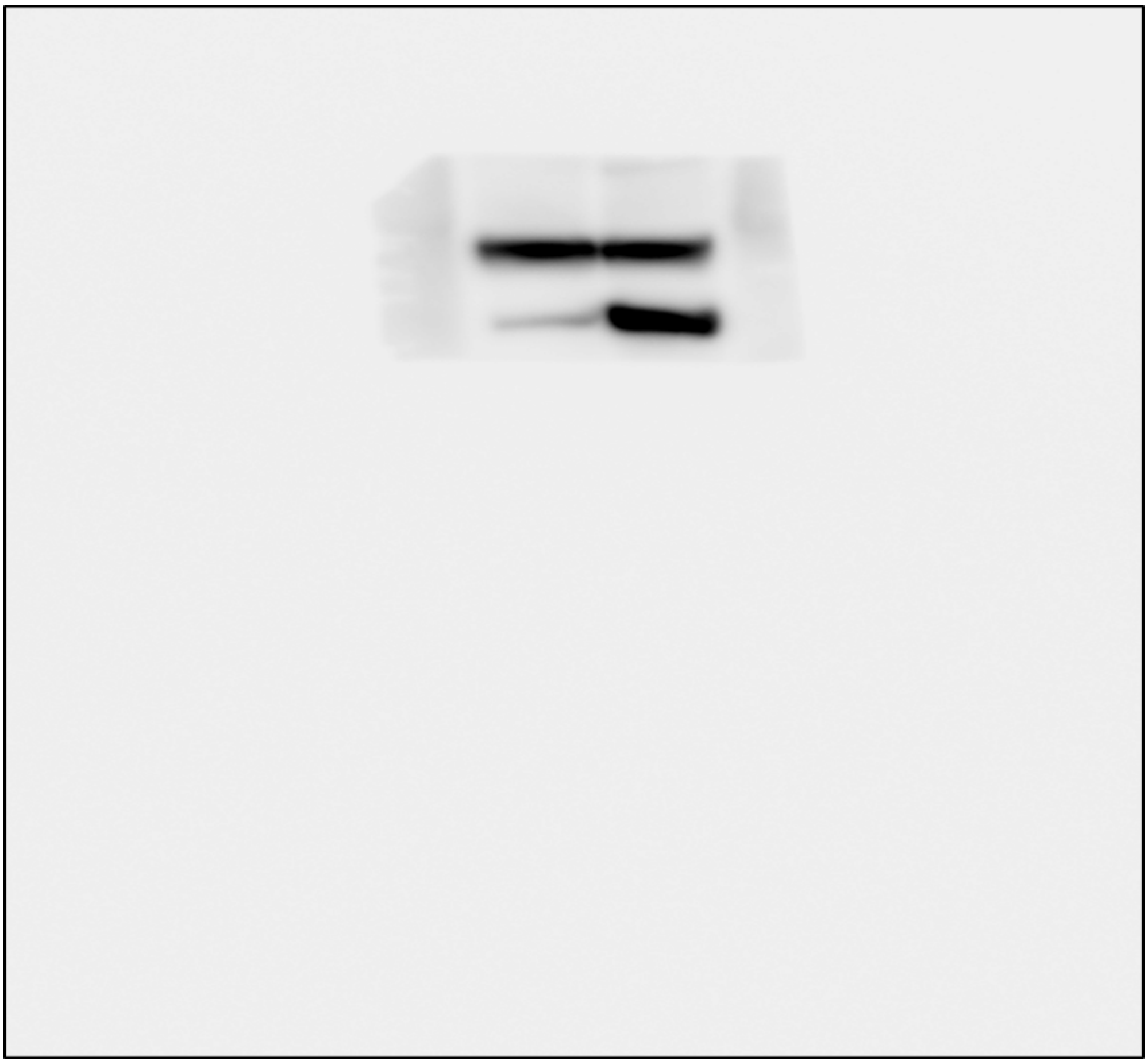

Fig-2D Tubulin

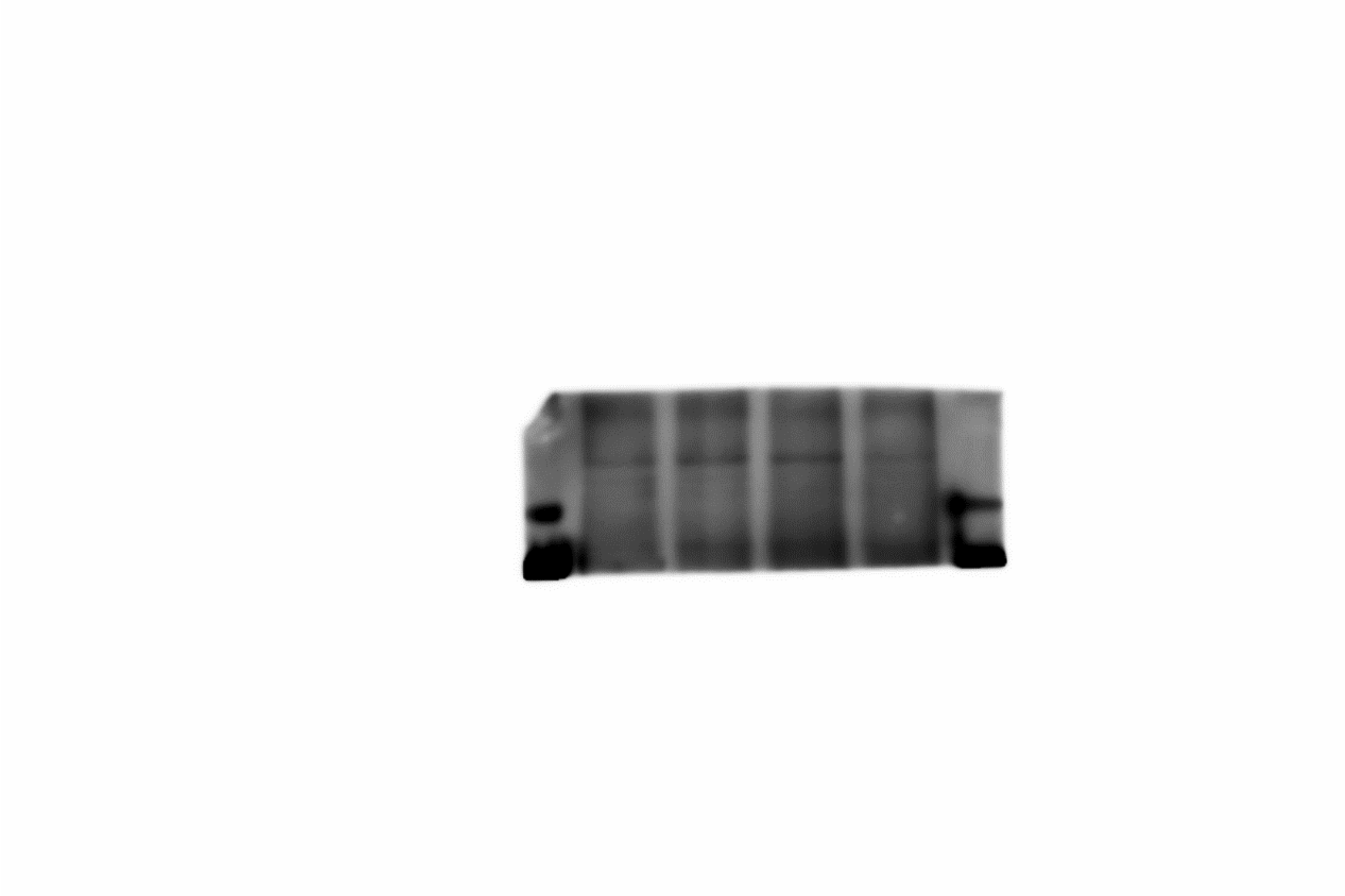

Fig-3C MUC16

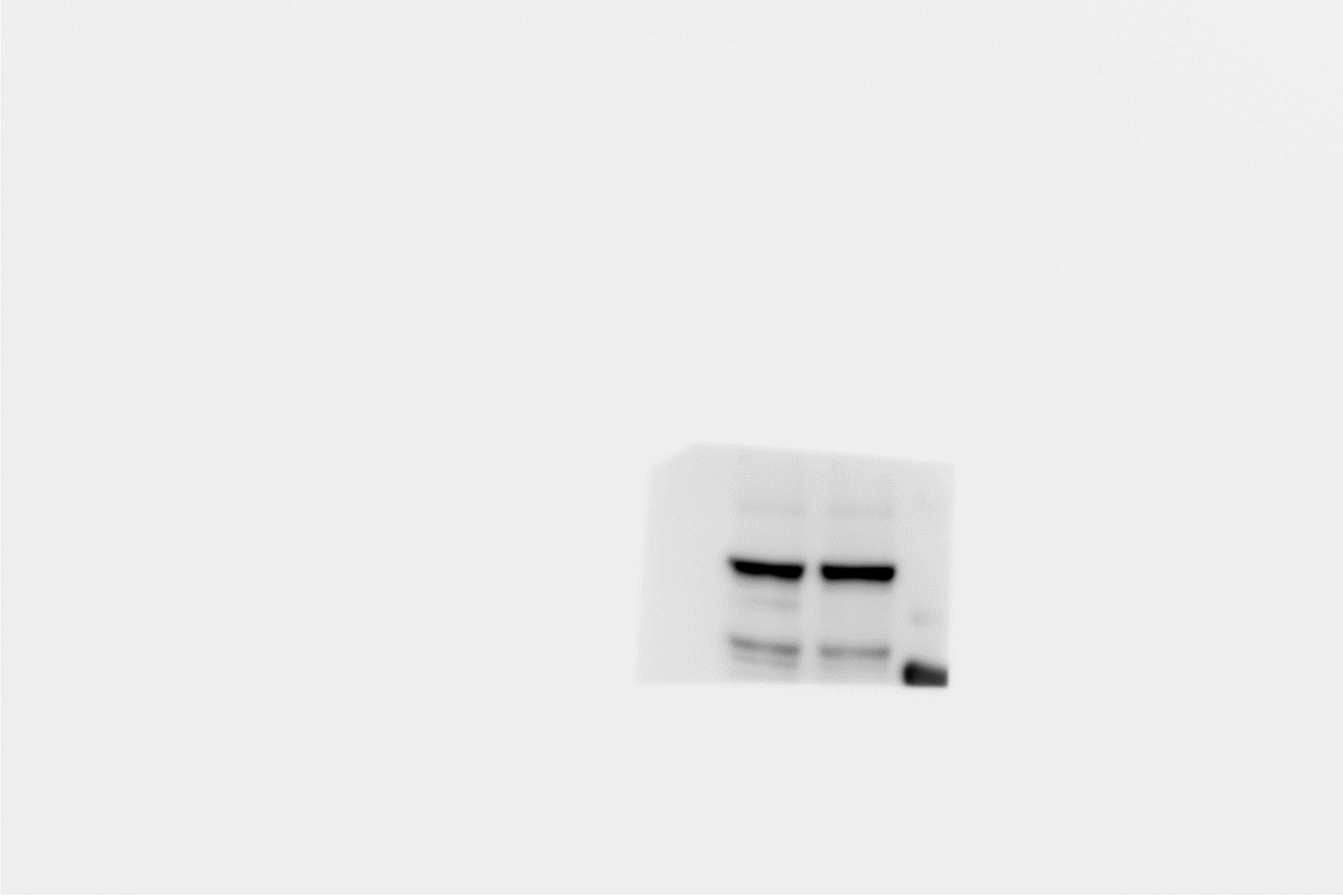

Fig-3C MUC16 input

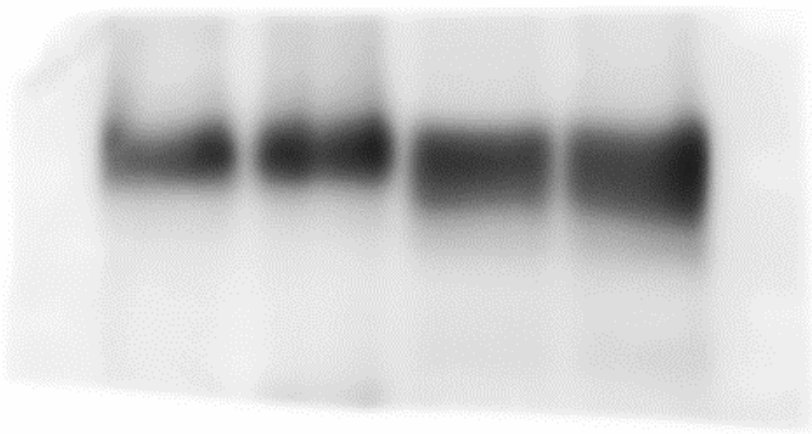

Fig-3C Siglec-7-1

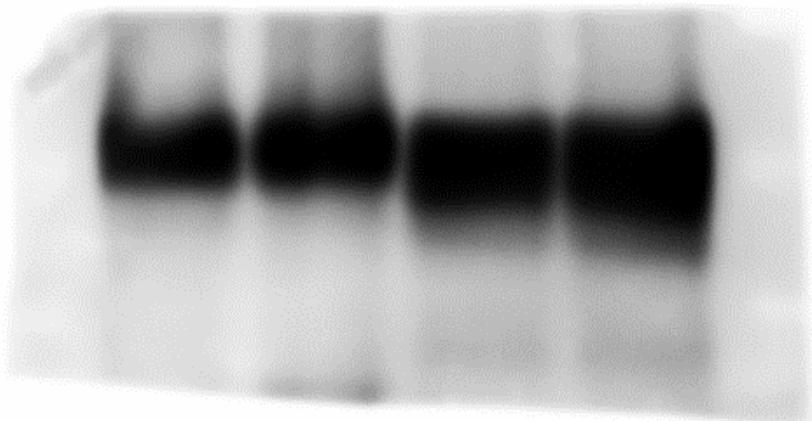

Fig-3C Siglec-7-2

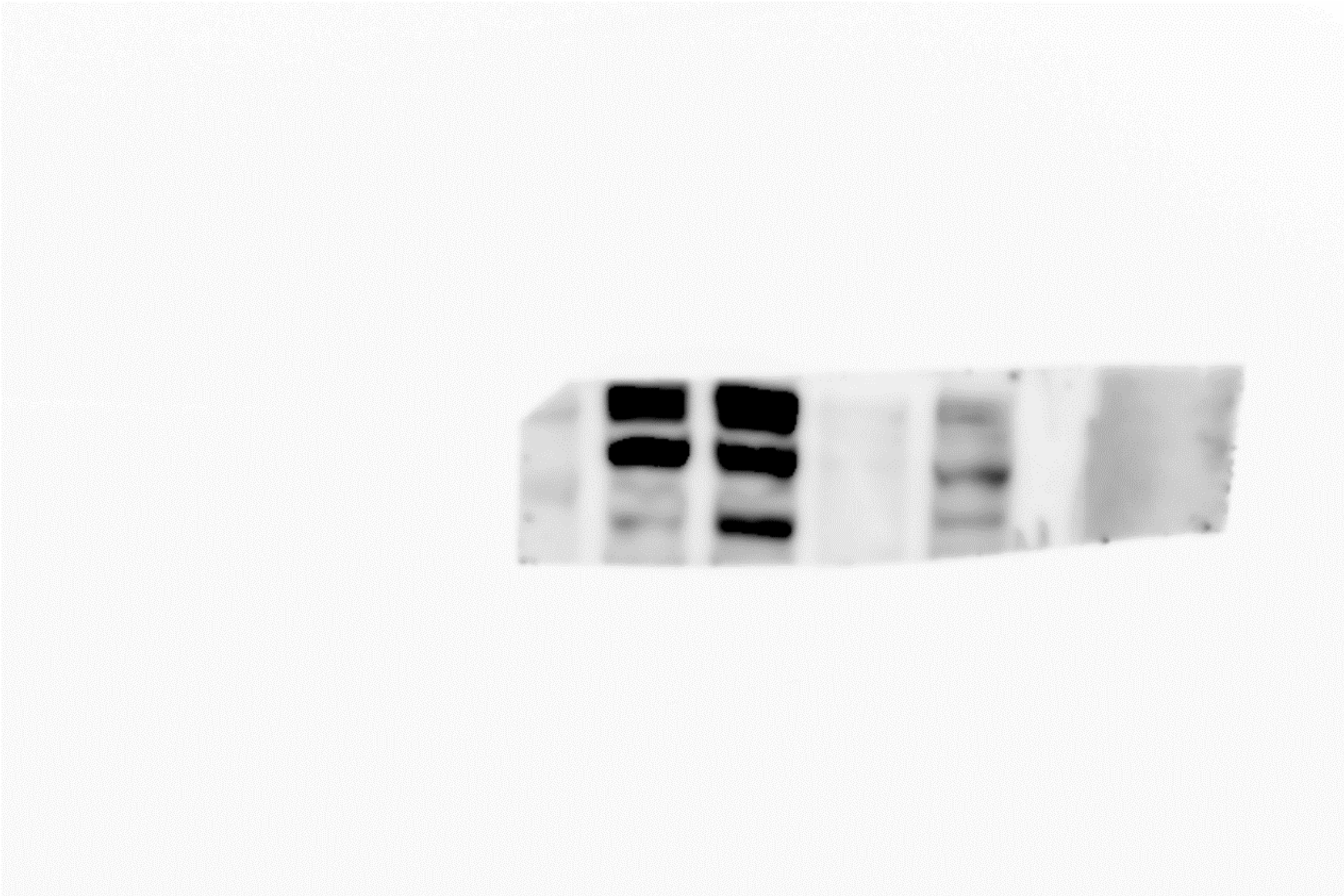

Fig-4A MUC16

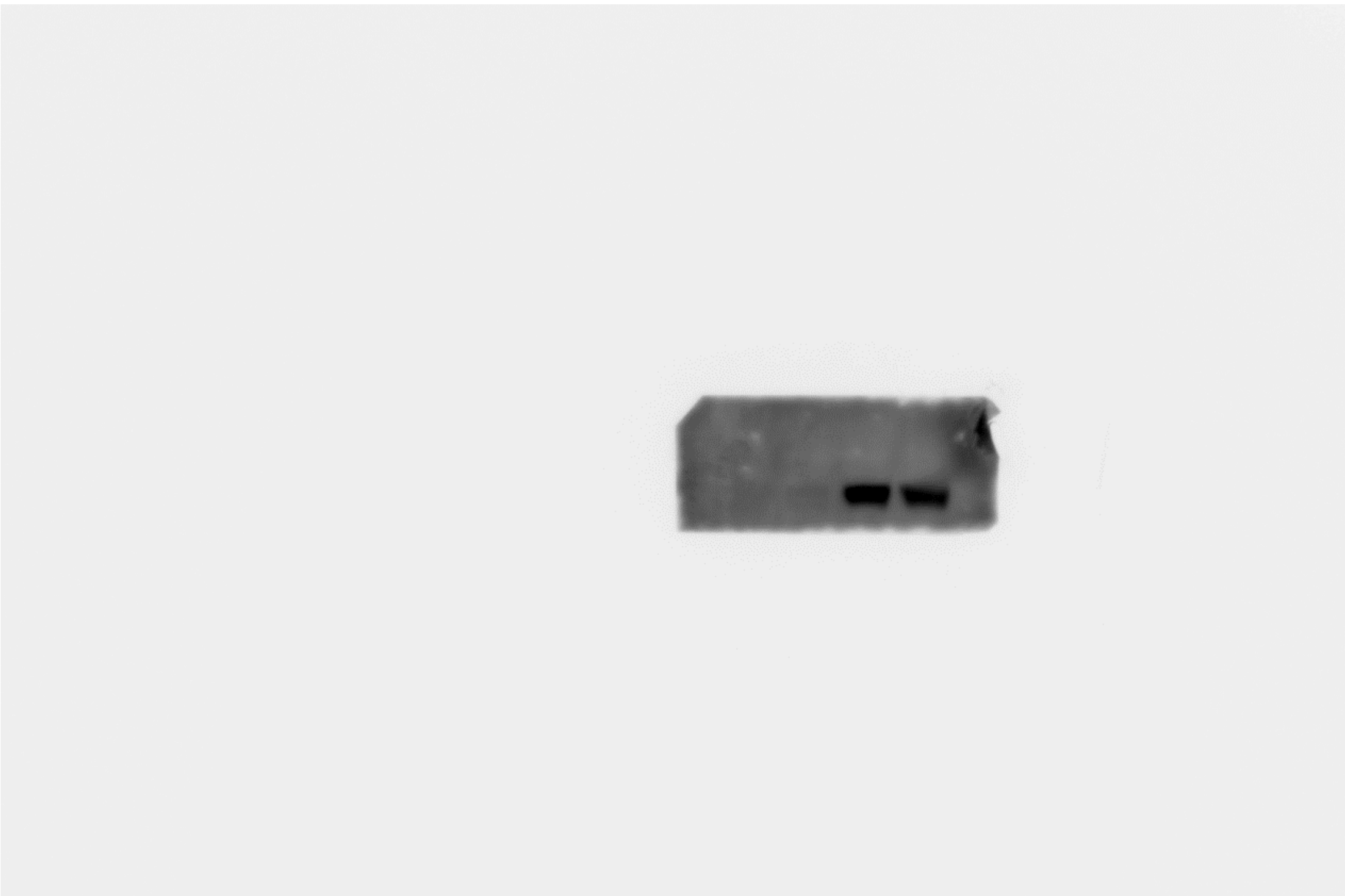

Fig-4A pan-cadherin

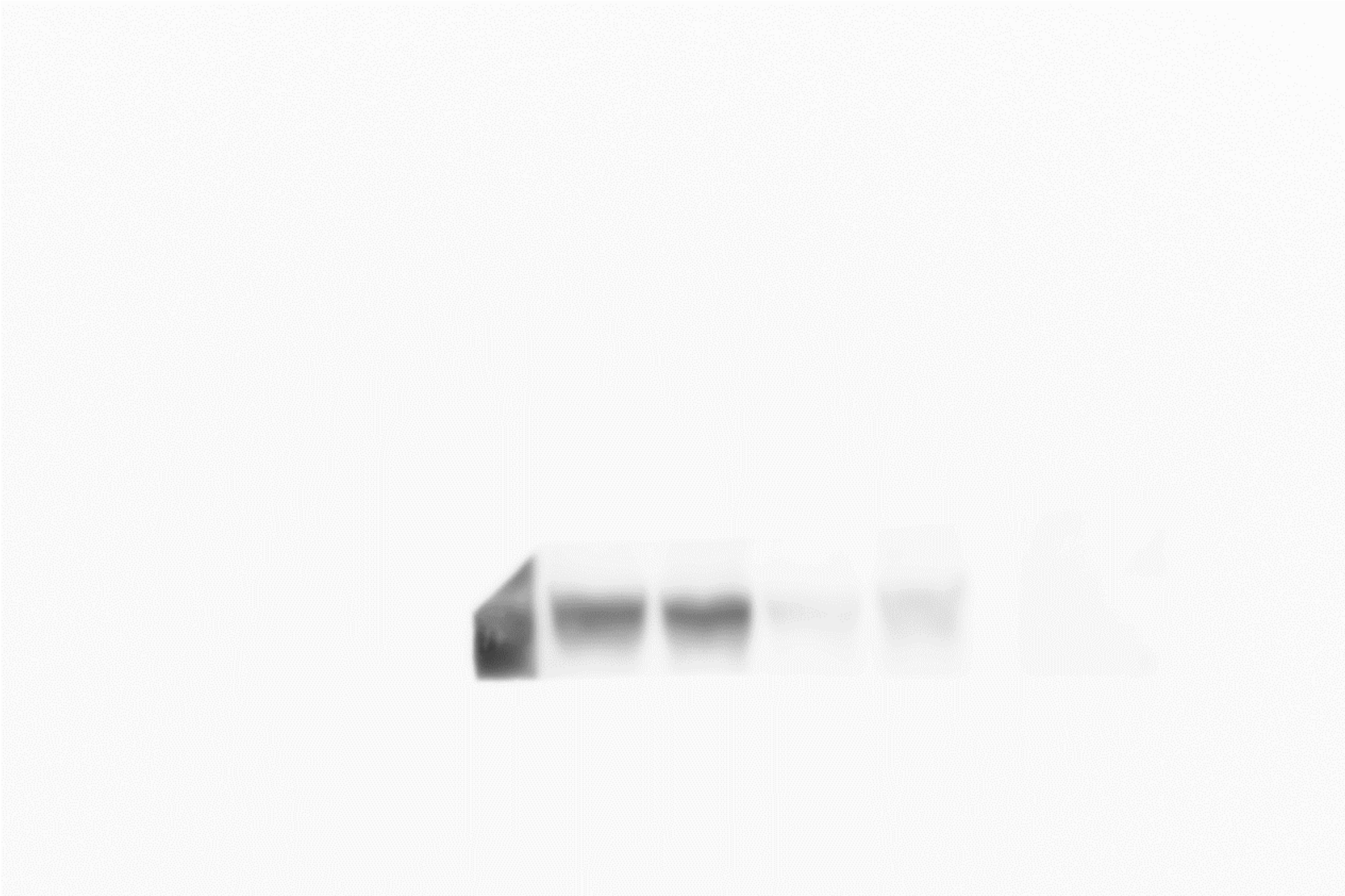

Fig-4A Tubulin

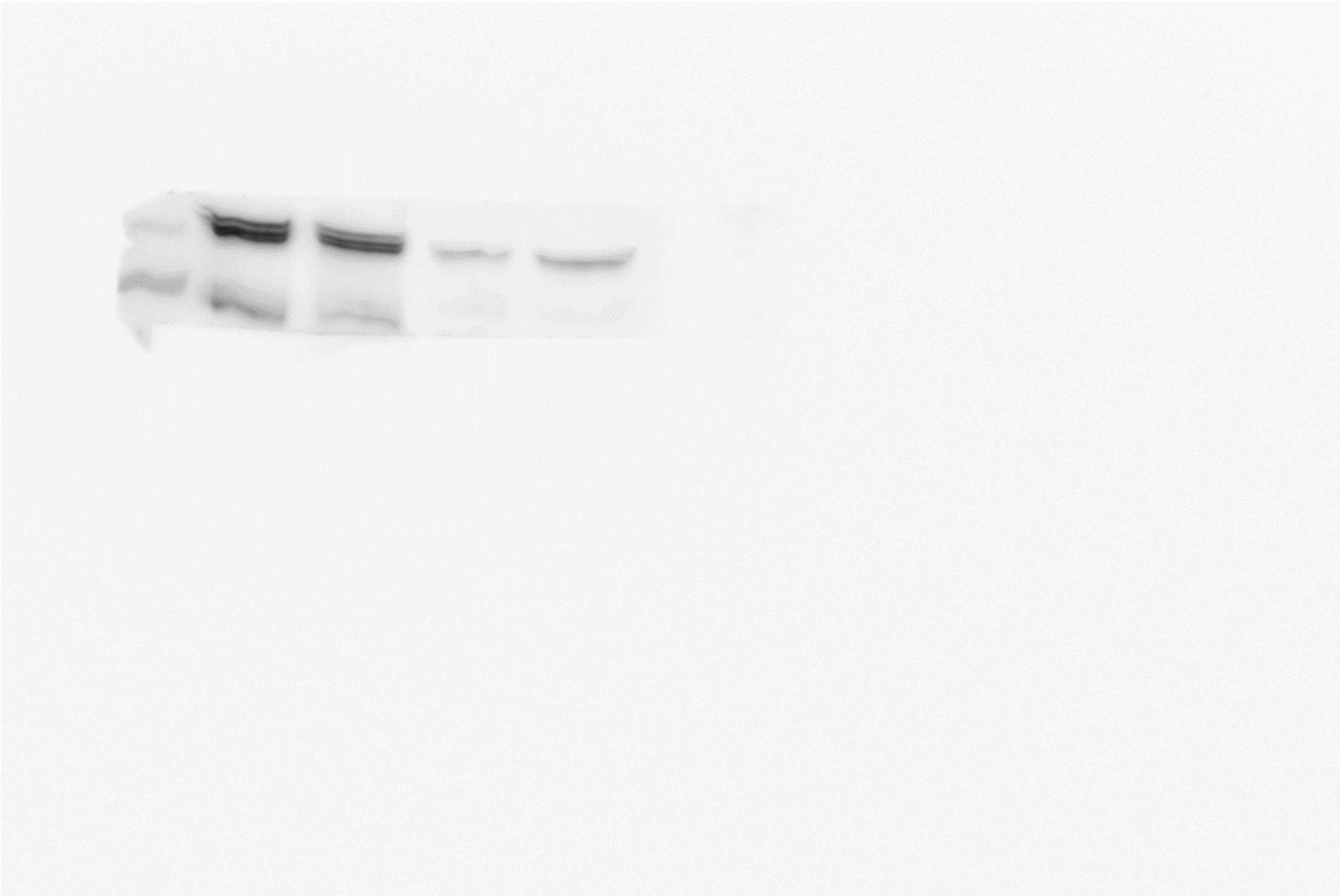

Fig-4B MUC16

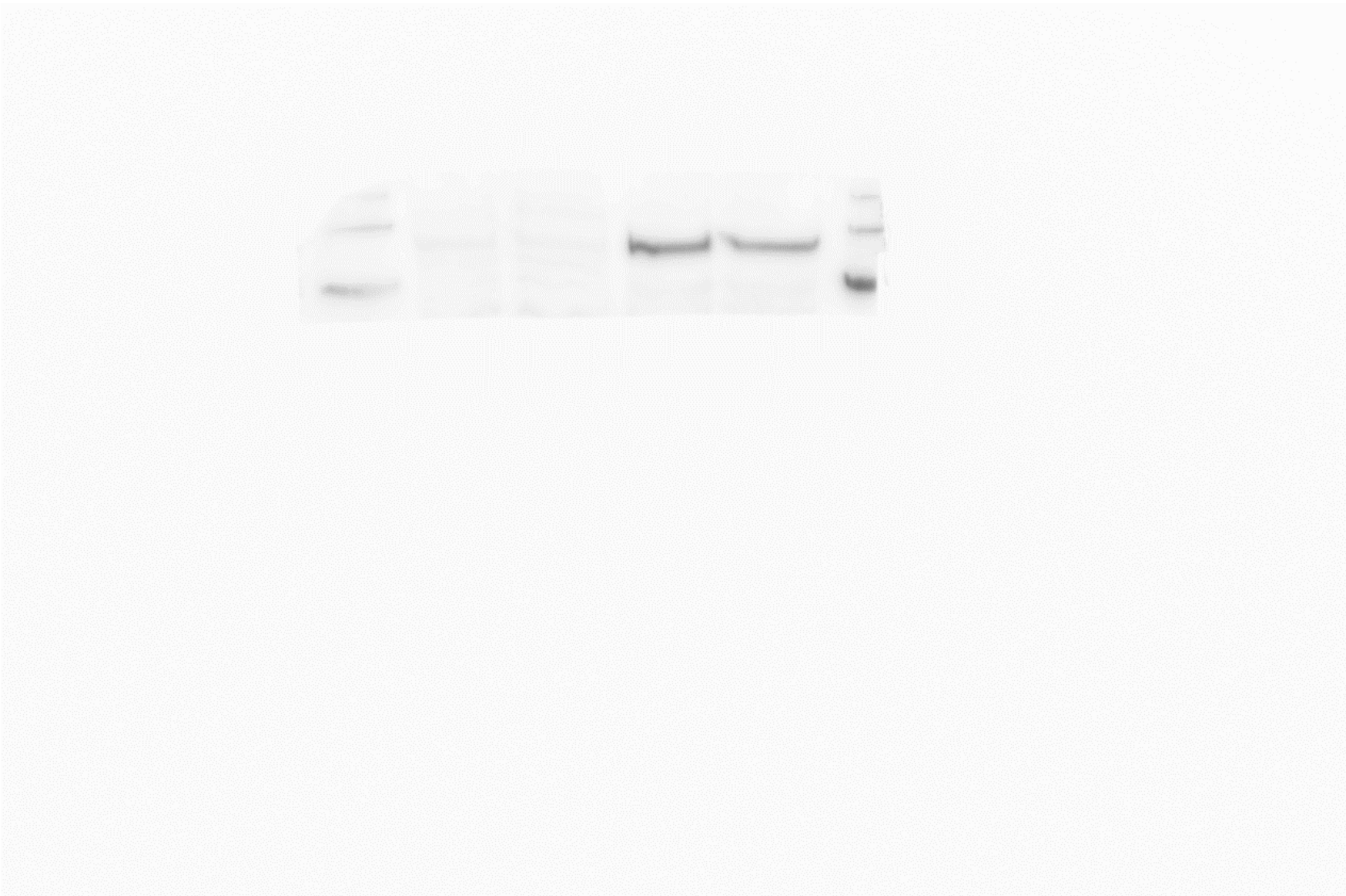

Fig-4B pan-cadherin

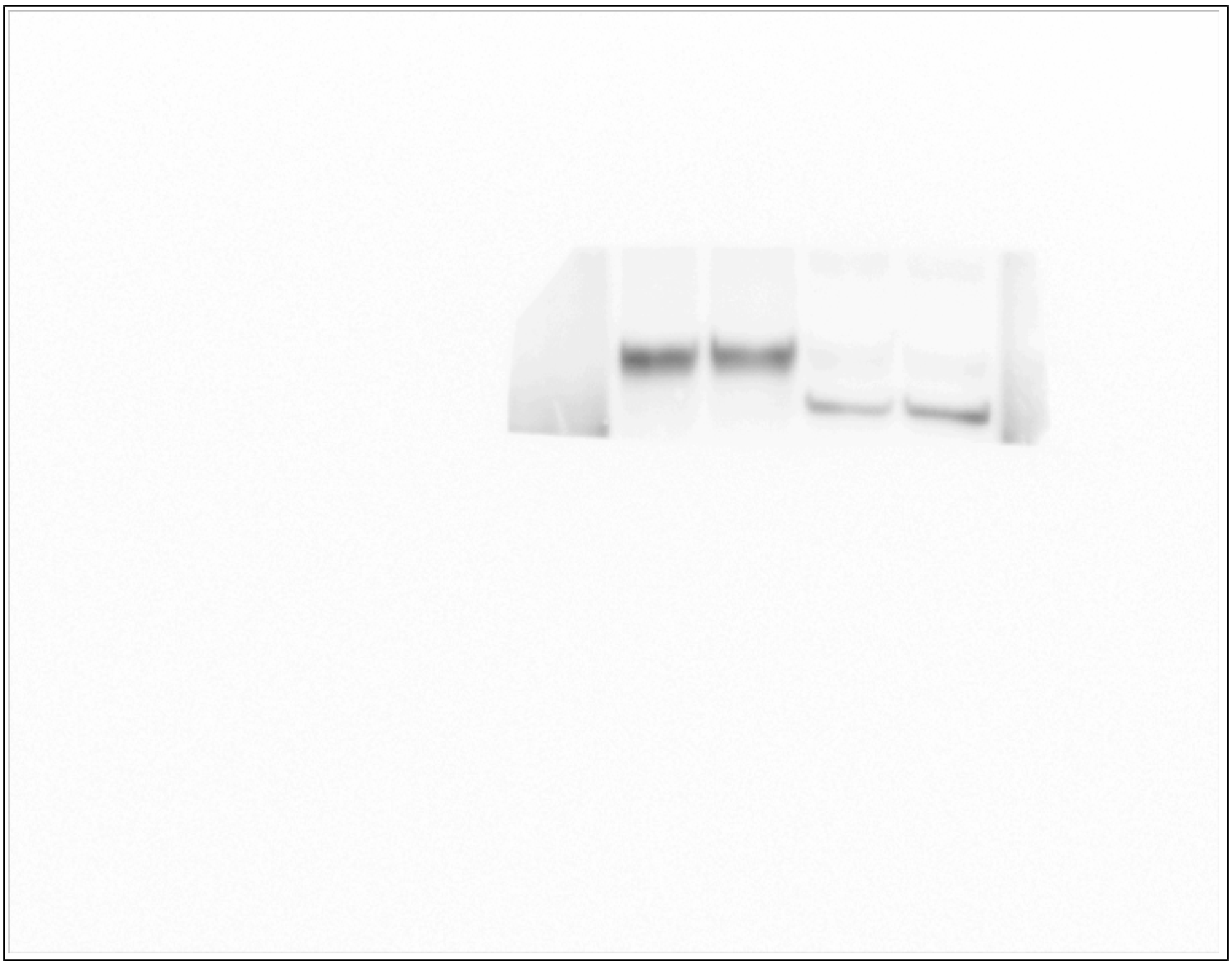

Fig-4B Tubulin

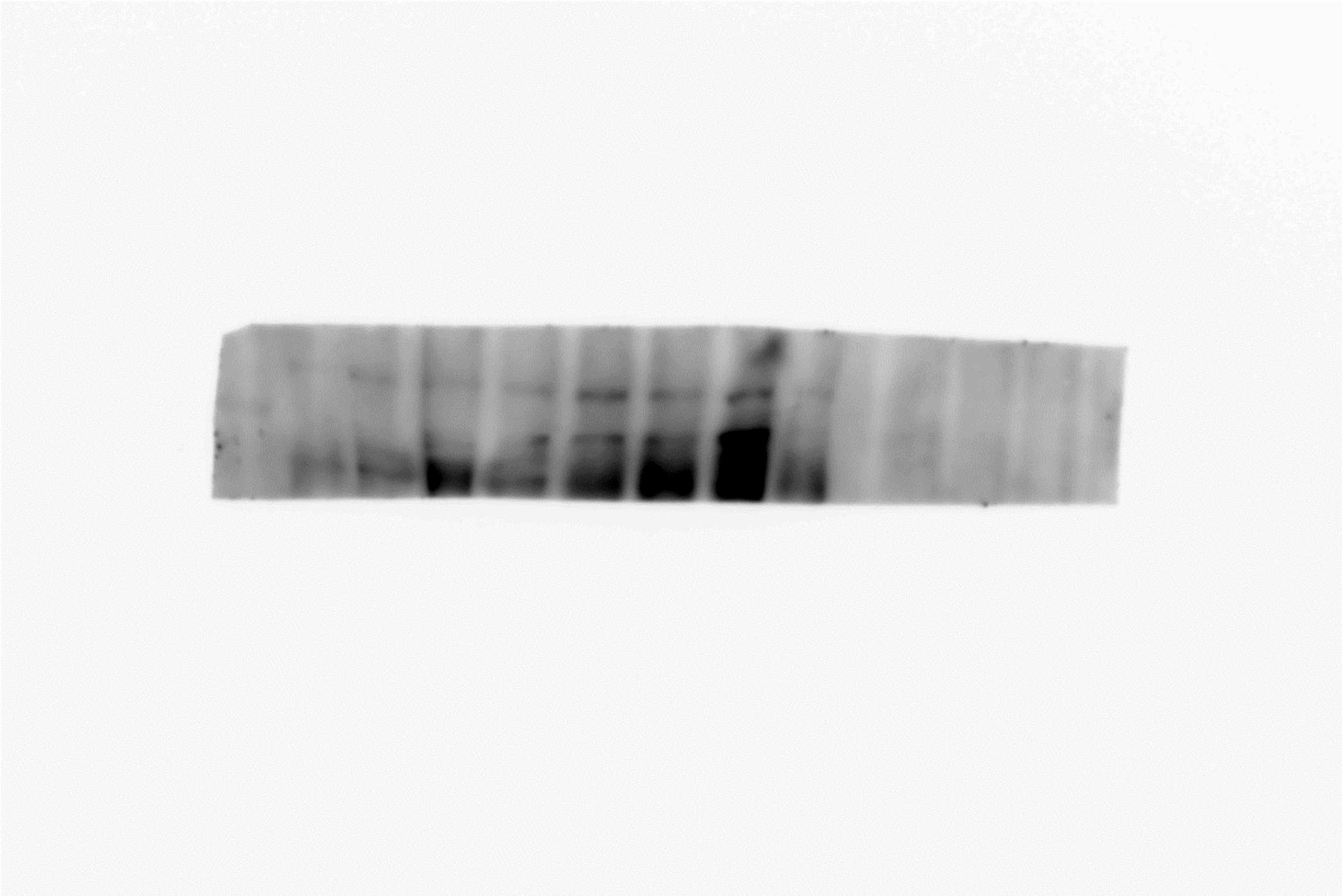

Fig-4E MUC16

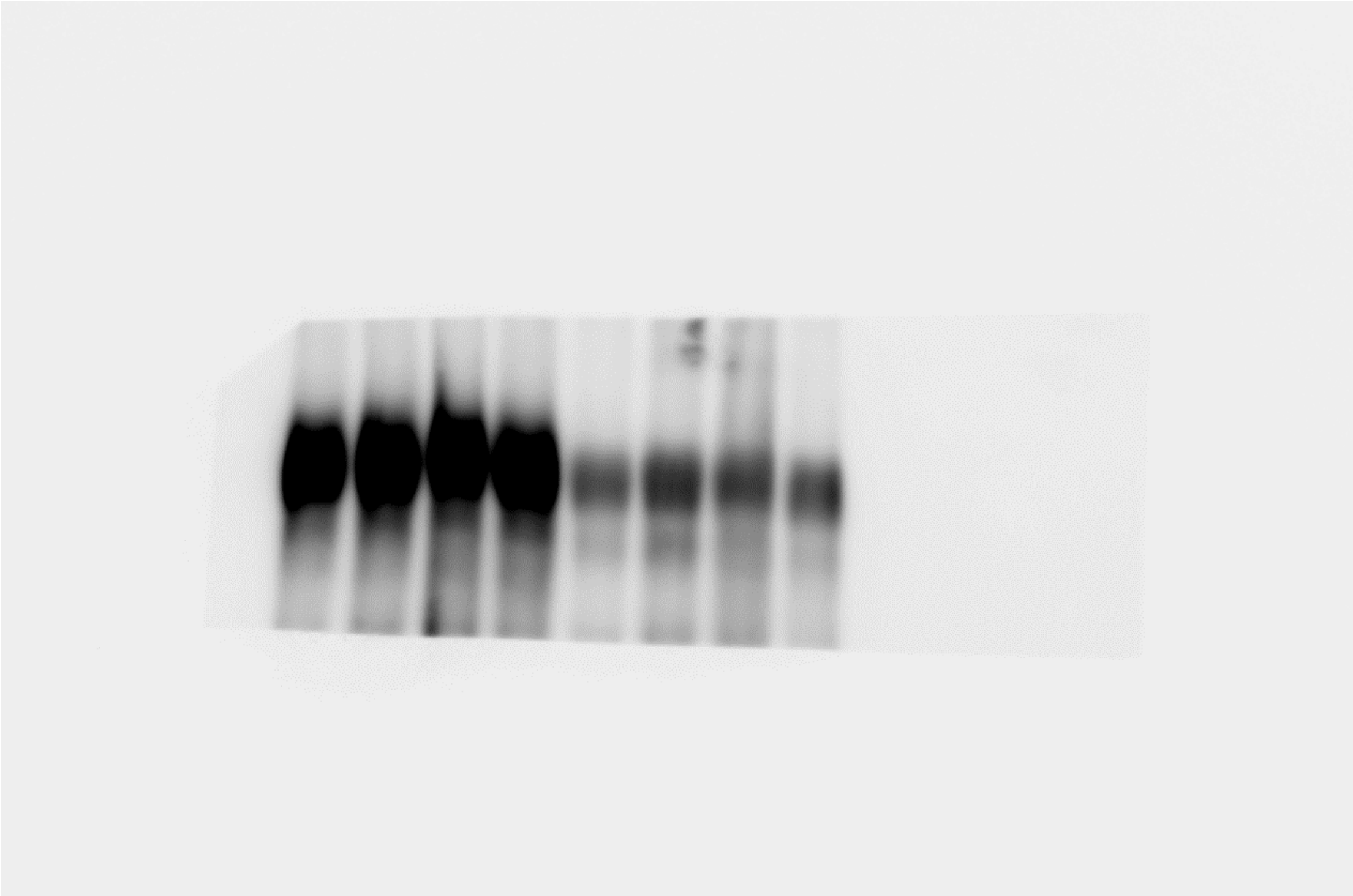

Fig-4E Siglec-7

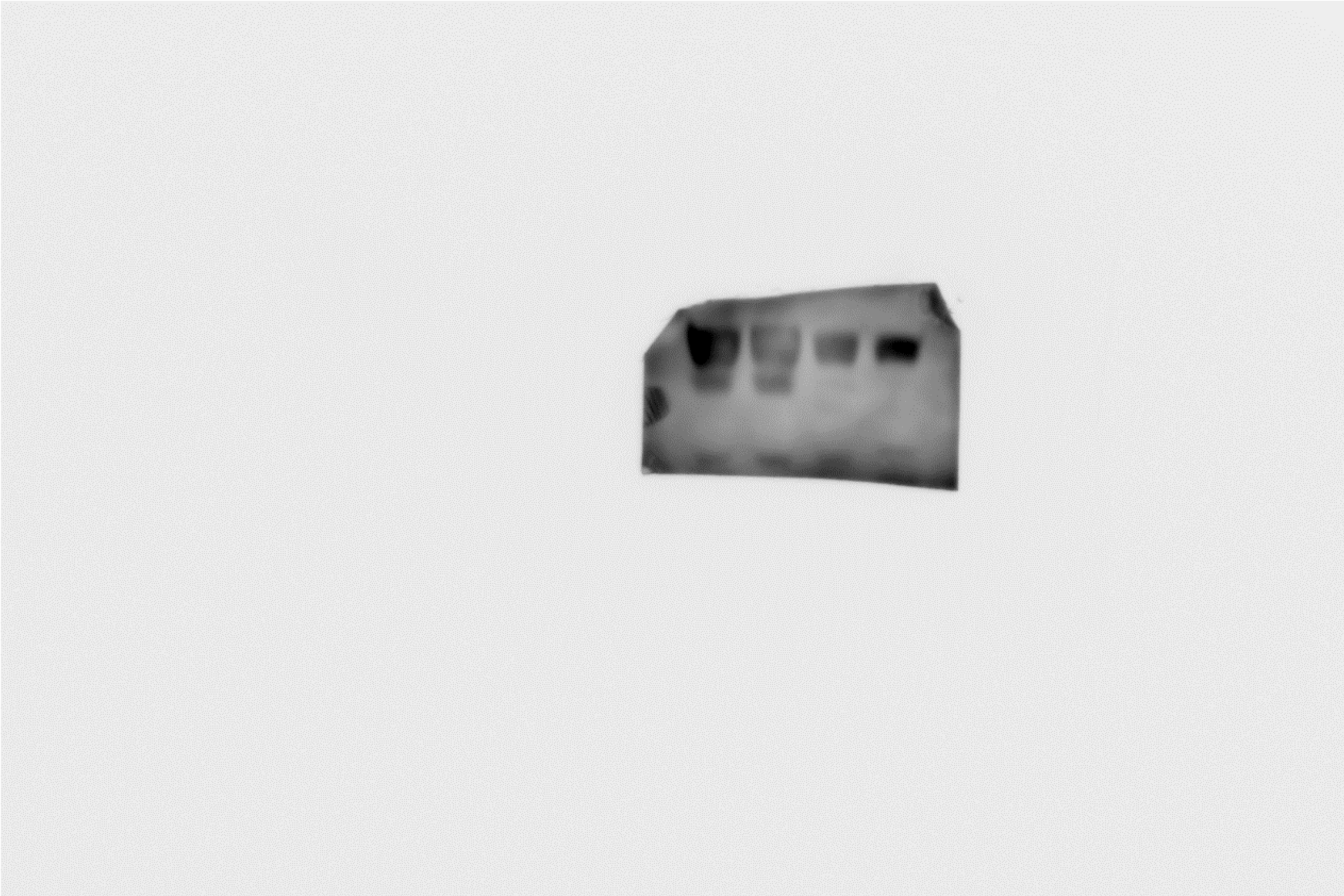

Fig-4E pan-cadherin

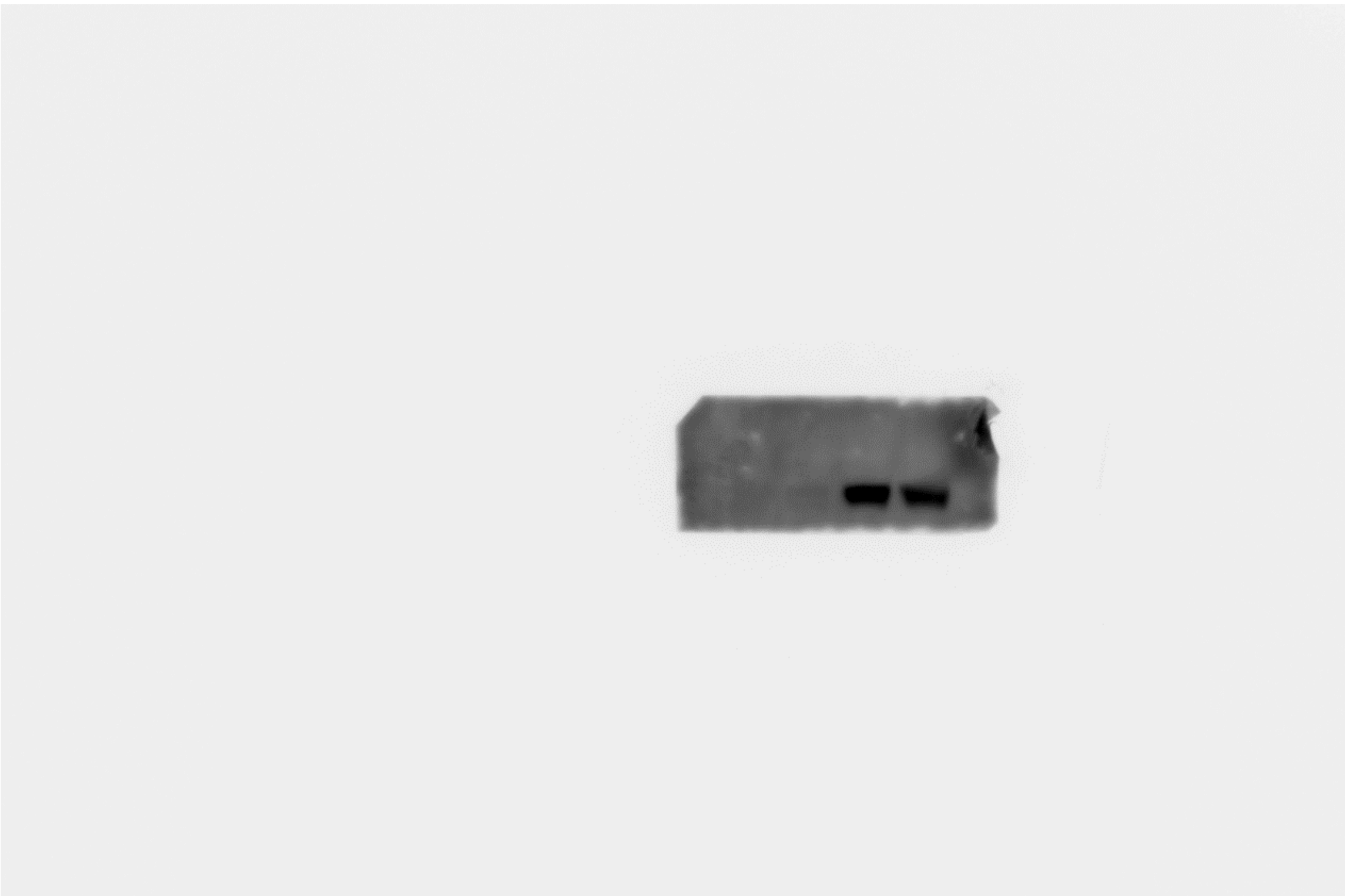

Fig-4E Tubulin

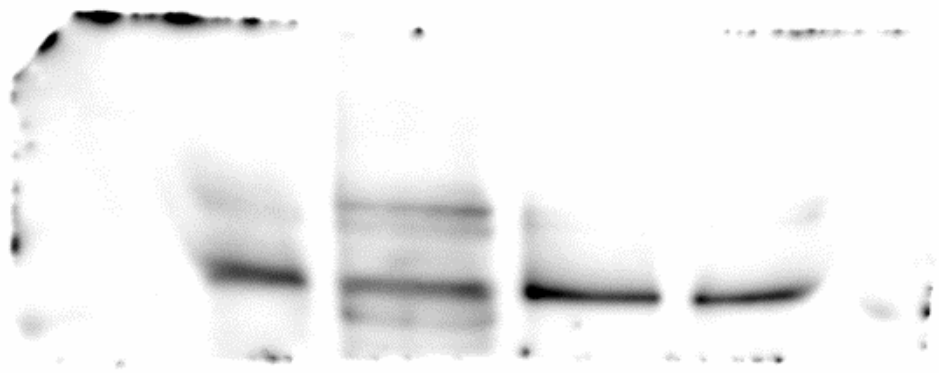

Fig-4F MUC16

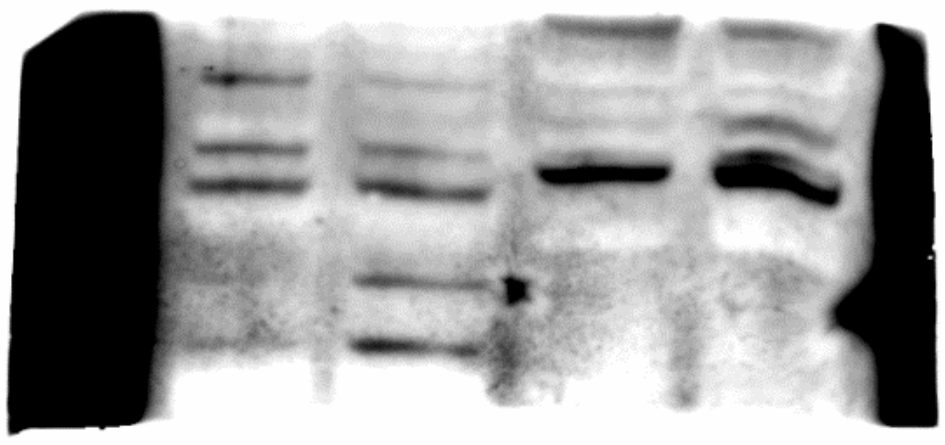

Fig-4F Siglec-7

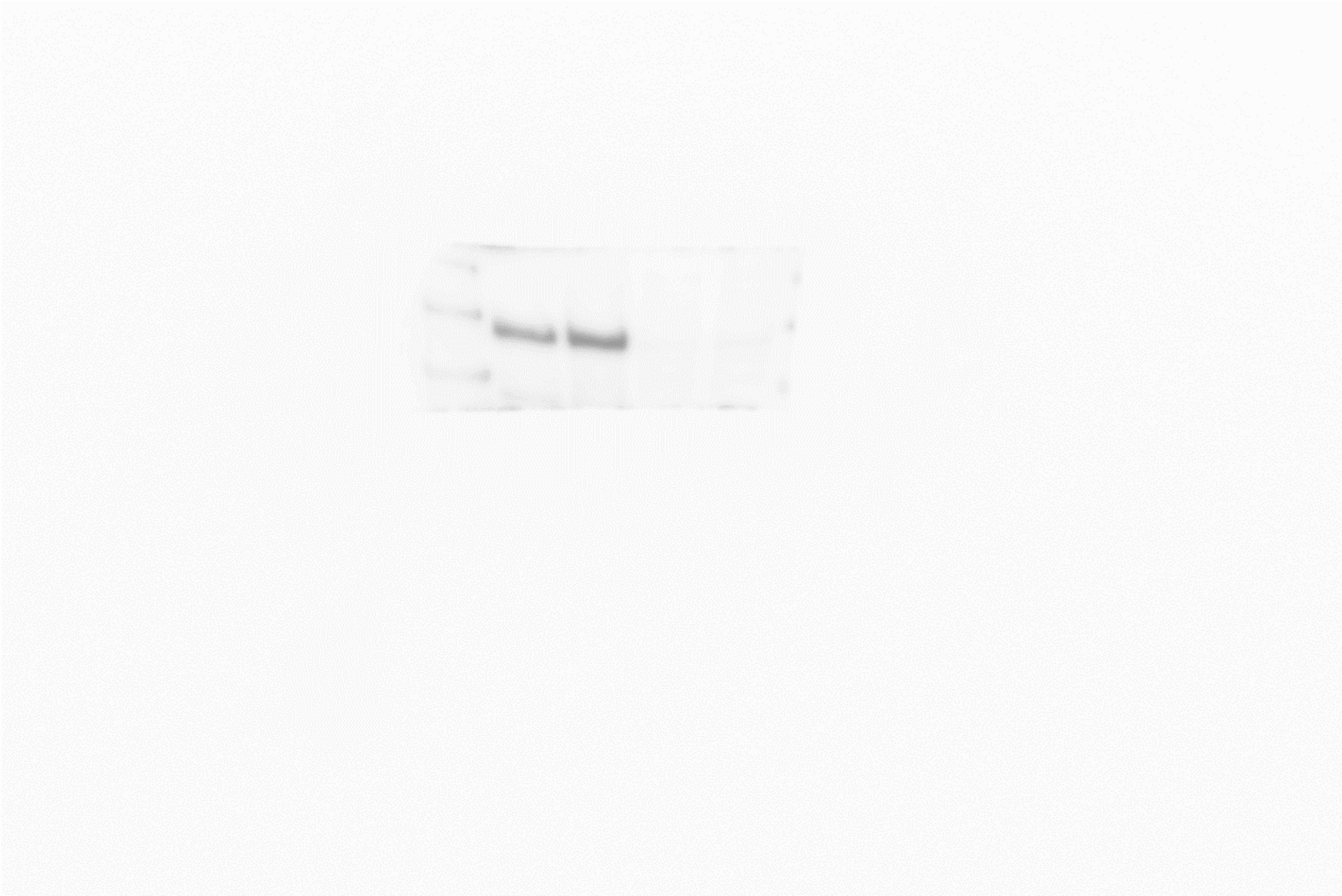

Fig-4F pan-cadherin

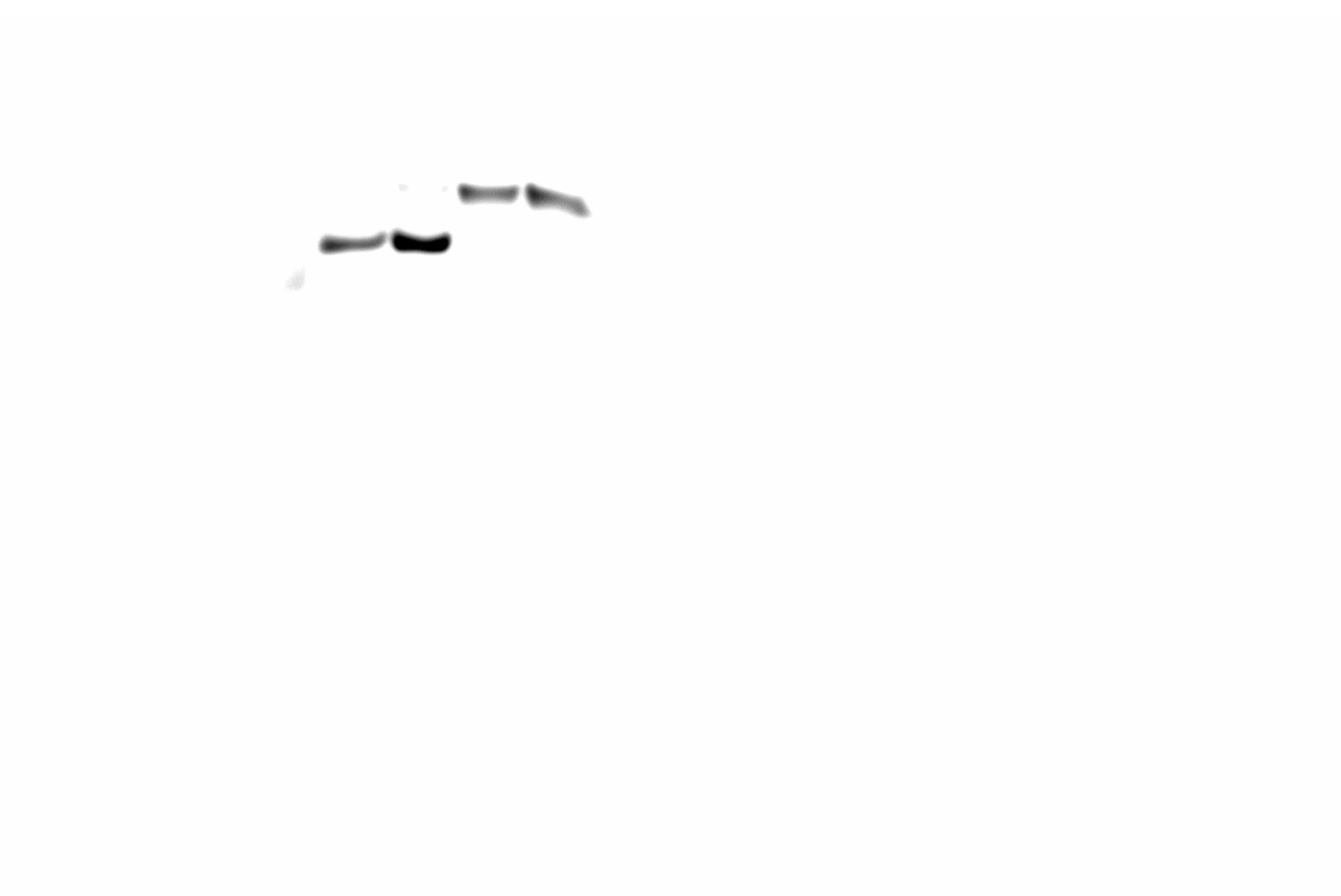

Fig-4F Tubulin

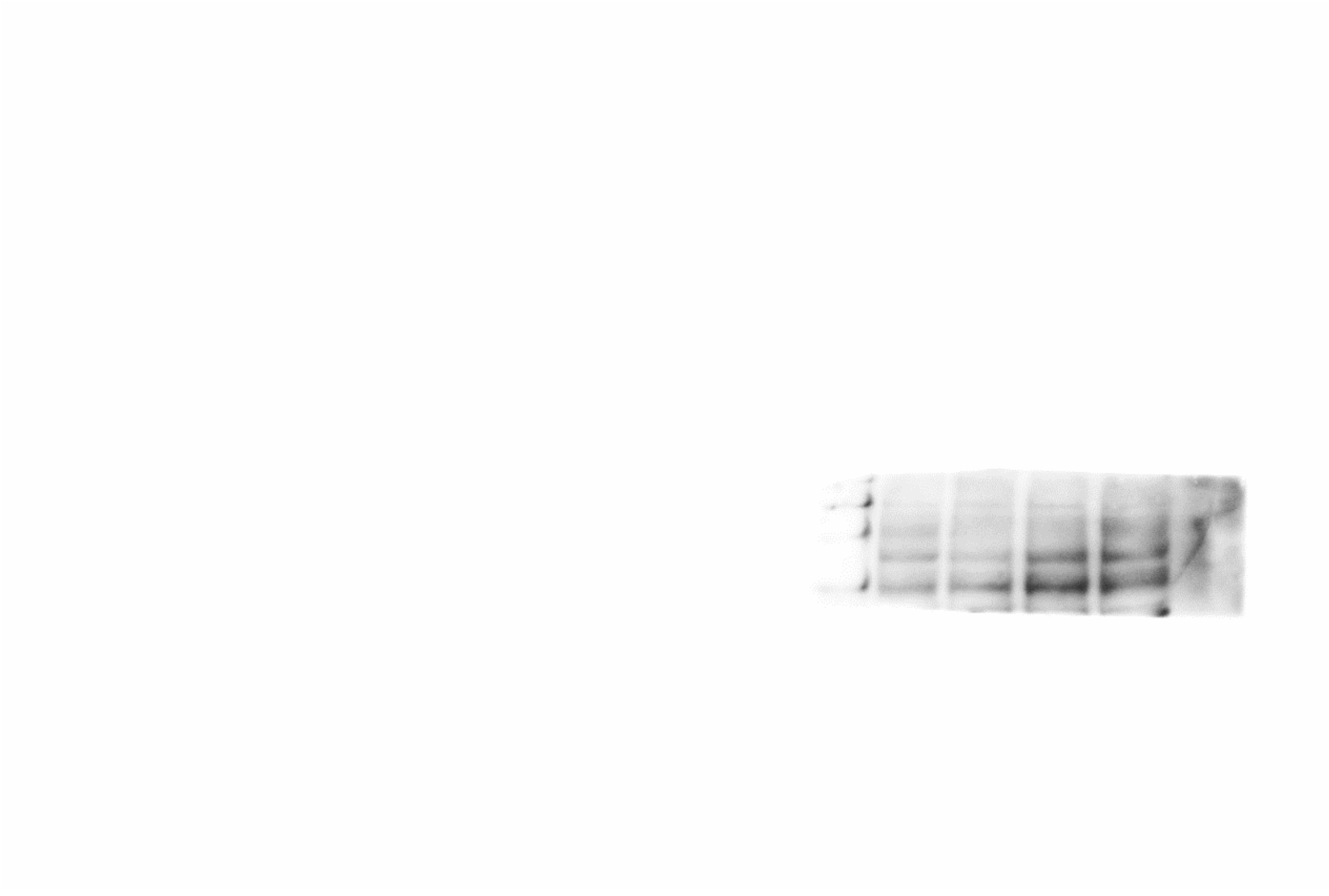

Fig-5A FAK-p576

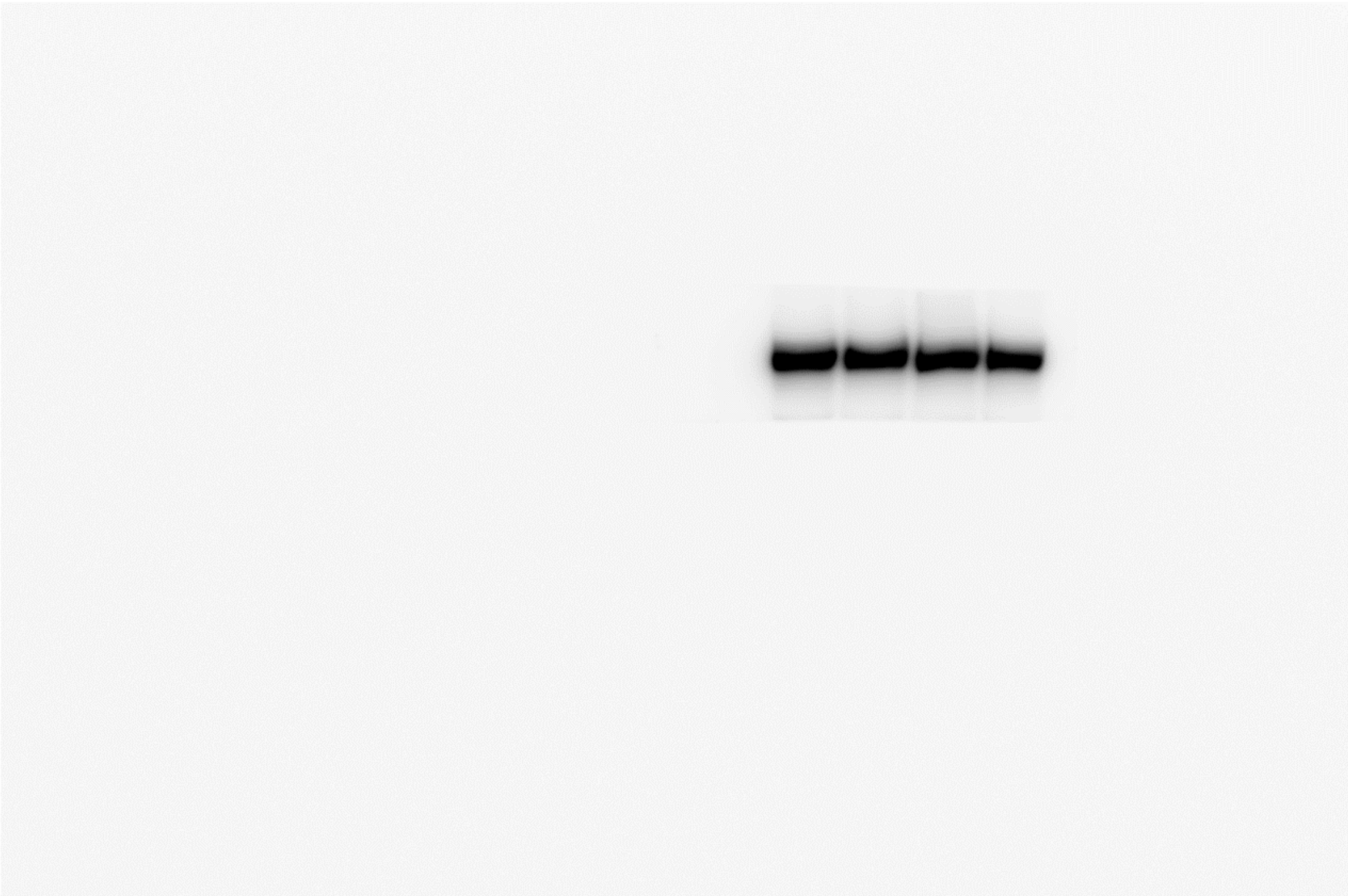

Fig-5A FAK

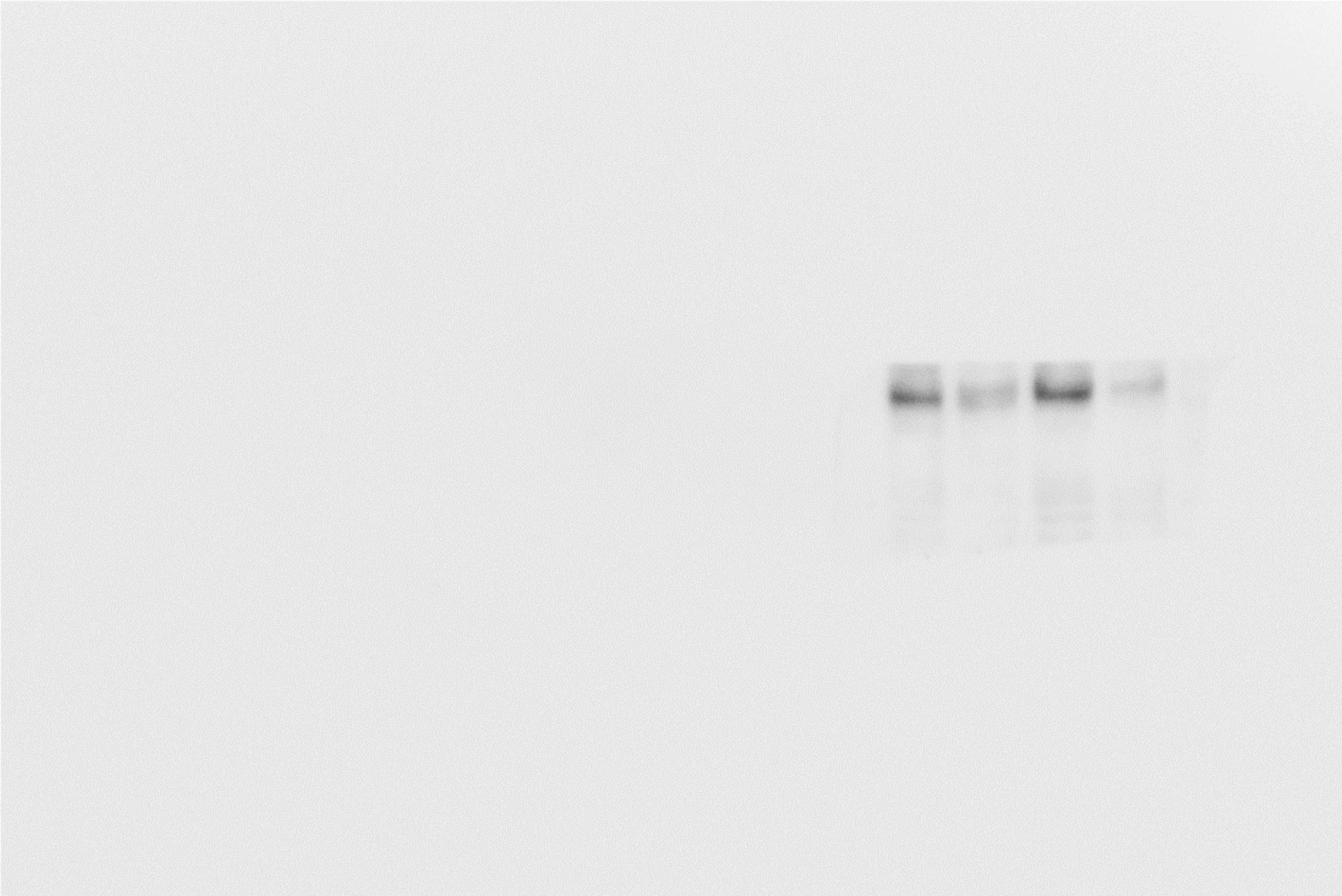

Fig-5A pSrc

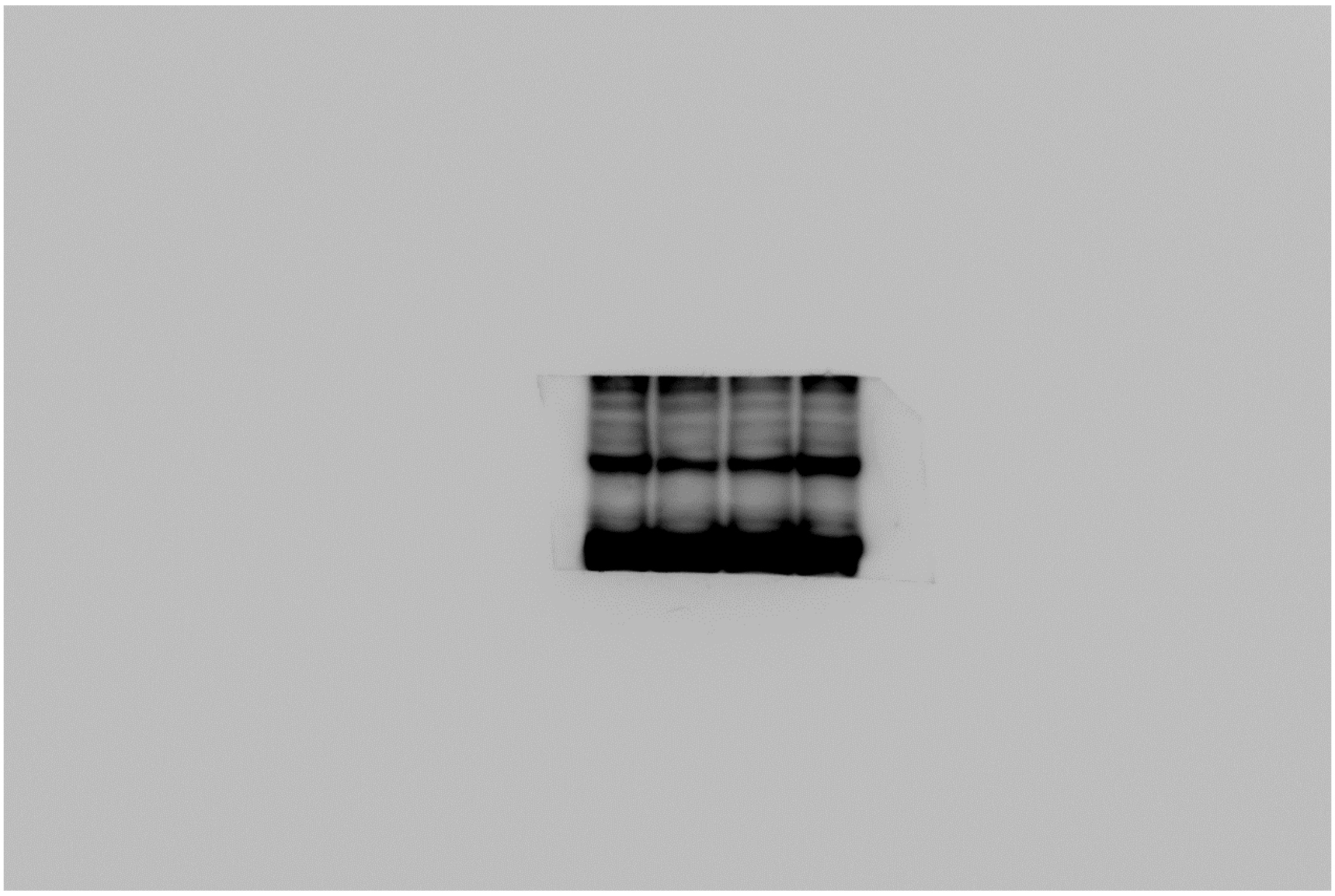

Fig-5A Src

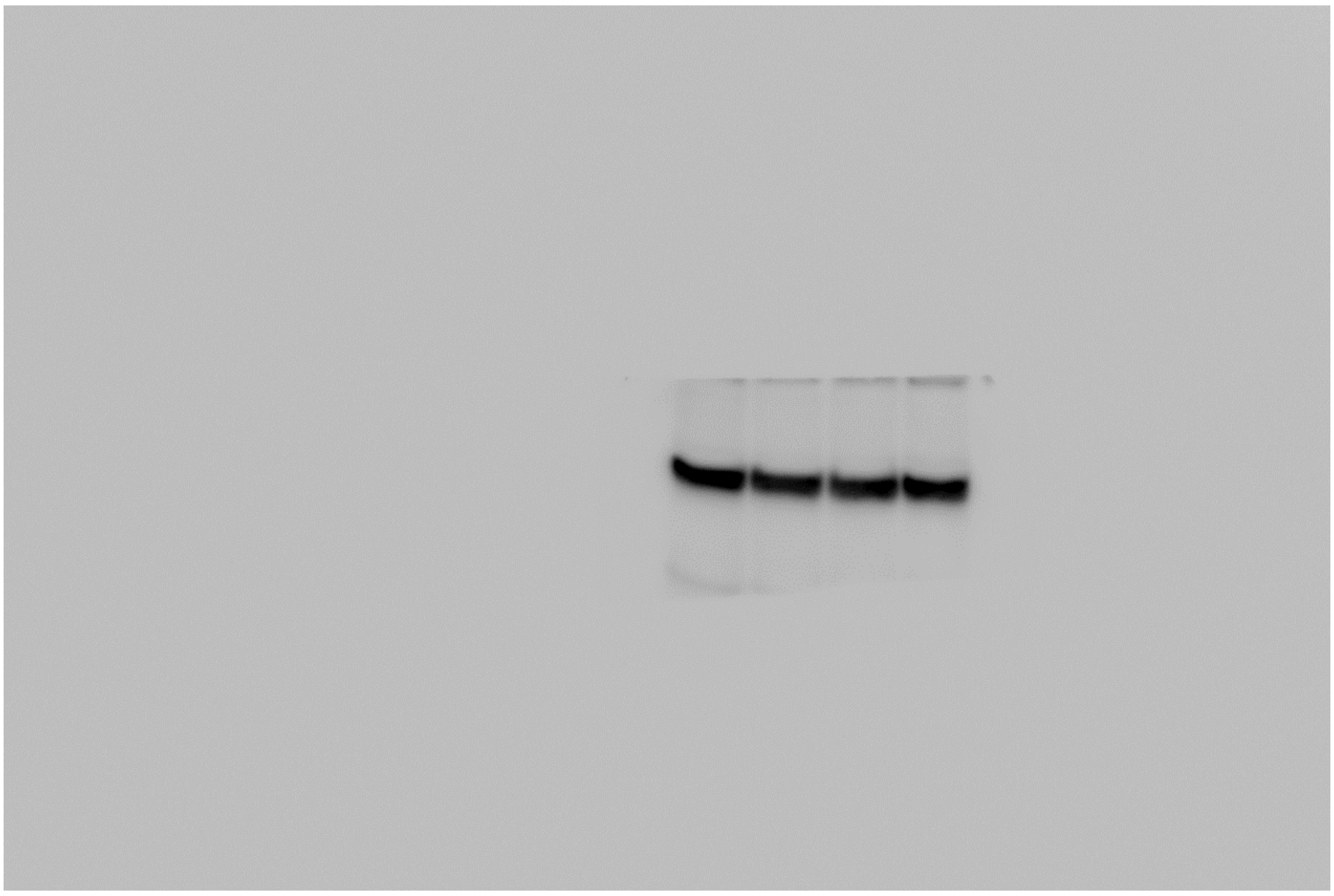

Fig-5A Tubulin

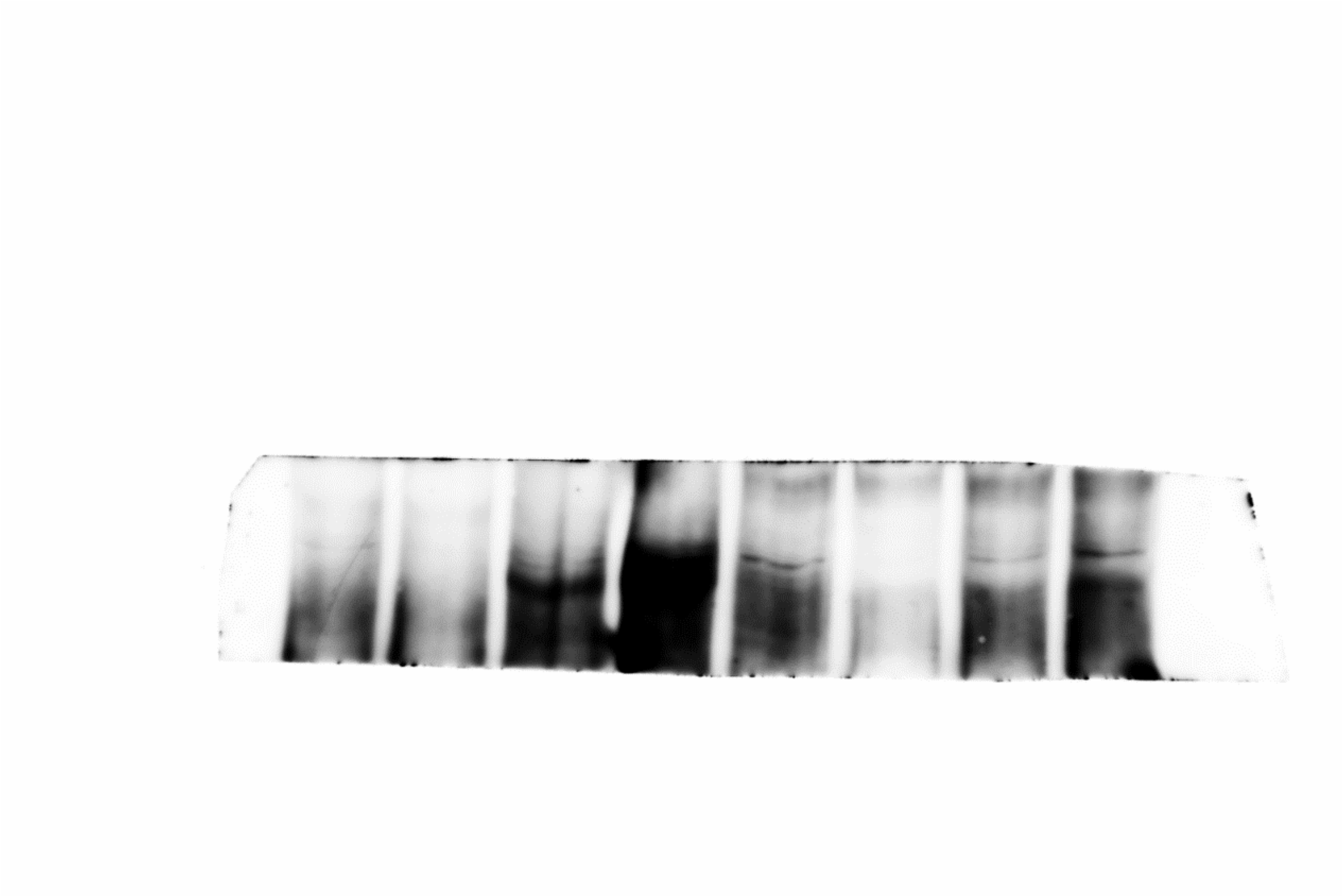

Fig-5B MUC16

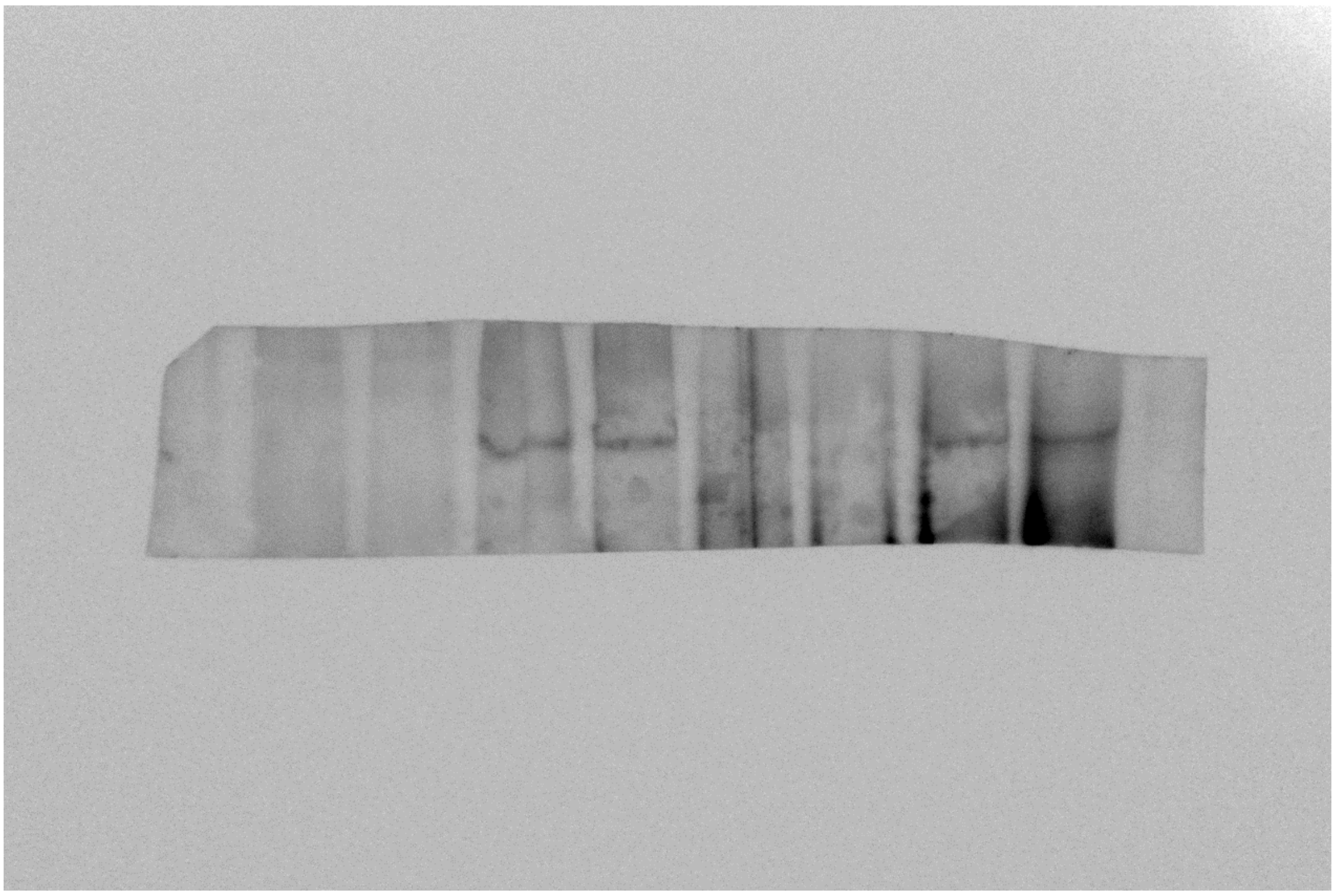

Fig-5B integrin β4

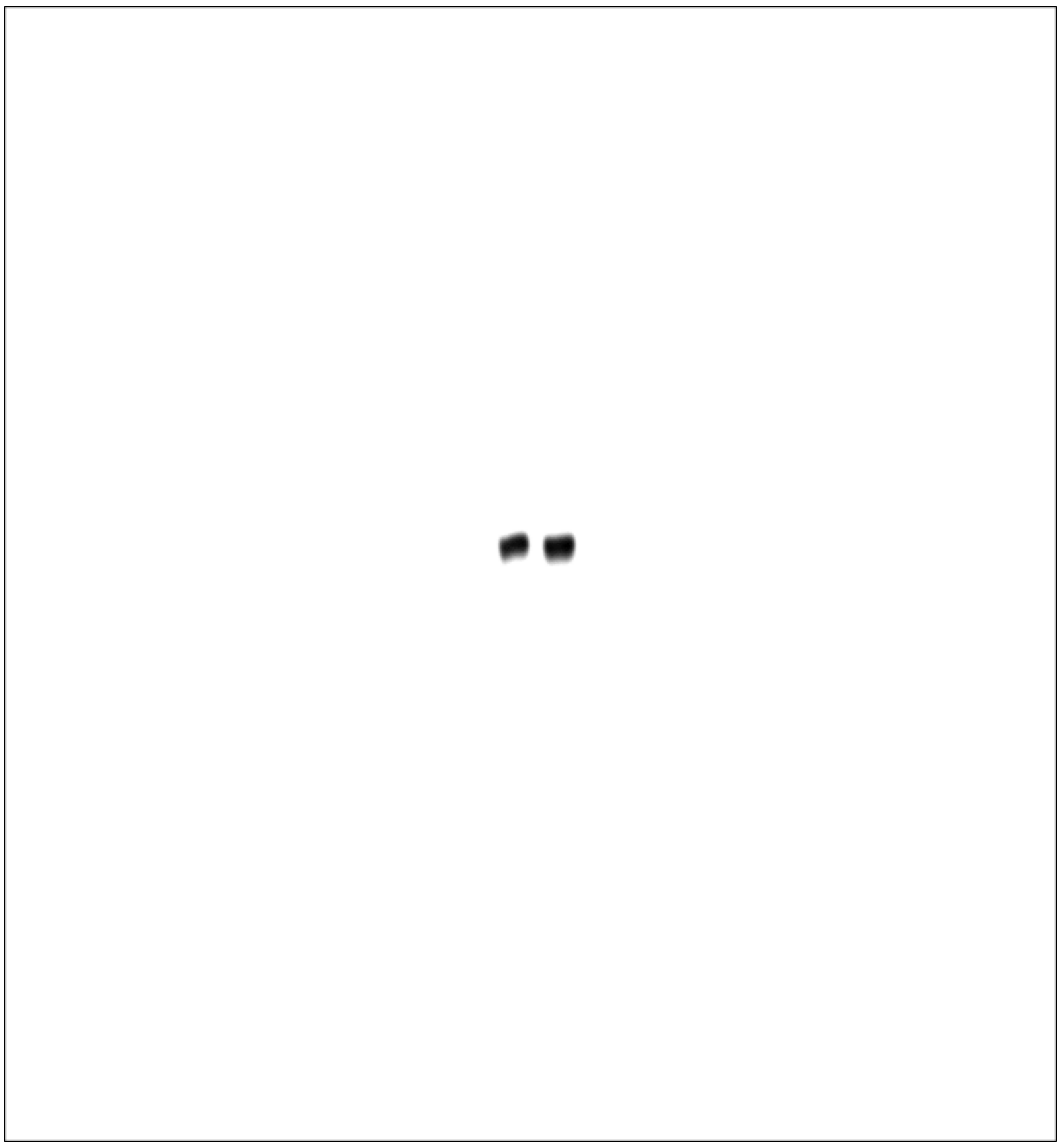

Fig-5B Tubulin

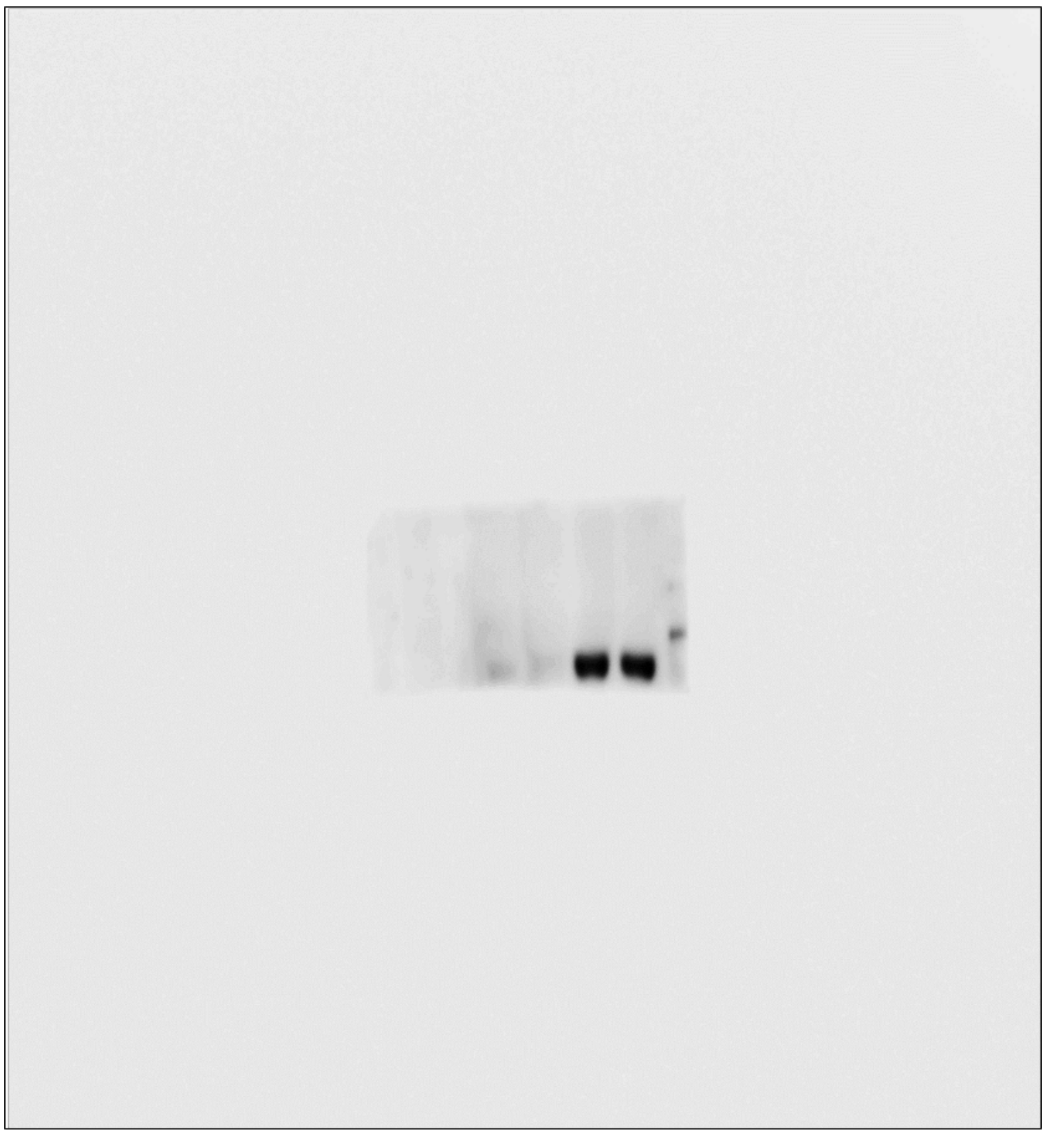

Fig-5B Pan-Cadherin
